# Manufacturing and preclinical toxicity of GLP grade gene deleted attenuated *Leishmania donovani* parasite vaccine

**DOI:** 10.1038/s41598-024-64592-6

**Published:** 2024-06-25

**Authors:** Kumar Avishek, Mirza A. Beg, Kavita Vats, Avinash Kumar Singh, Ranadhir Dey, Kamaleshwar P. Singh, Rajesh Kumar Singh, Sreenivas Gannavaram, V. Ramesh, Mohmad Sadik A. Mulla, Upendra Bhatnagar, Sanjay Singh, Hira L. Nakhasi, Poonam Salotra, Angamuthu Selvapandiyan

**Affiliations:** 1 Department of Molecular Medicine, Jamia Hamdard, New Delhi, 110062 India; 2 ICMR-National Institute of Pathology, Safdarjung Hospital Campus, New Delhi, 110029 India; 3 Division of Emerging and Transfusion Transmitted Diseases, CBER, Food and Drug Administration, Silver Spring, MD 20993 USA; 4 Gennova Biopharmaceuticals, Hinjewadi Phase II, Pune, Maharashtra 411057 India; 5 Department of Dermatology and STD, ESIC Medical College, Faridabad, Haryana 121001 India; 6 Vimta Laboratories, Cherlapally, Hyderabad, Telangana 500051 India

**Keywords:** *Leishmania donovani*, Visceral leishmaniasis, Vaccine candidate, Live attenuated, Preclinical, Toxicity study, Immunology, Microbiology, Molecular medicine

## Abstract

Centrin1 gene deleted *Leishmania donovani* parasite (*LdCen1*^*−/−*^) was developed and extensively tested experimentally as an intracellular stage-specific attenuated and immunoprotective live parasite vaccine candidate ex vivo using human PBMCs and in vivo in animals. Here we report manufacturing and pre-clinical evaluation of current Good-Laboratory Practice (cGLP) grade *LdCen1*^*−/−*^ parasites, as a prerequisite before proceeding with clinical trials. We screened three batches of *LdCen1*^*−/−*^ parasites manufactured in bioreactors under cGLP conditions, for their consistency in genetic stability, attenuation, and safety. One such batch was preclinically tested using human PBMCs and animals (hamsters and dogs) for its safety and protective immunogenicity. The immunogenicity of the CGLP grade *LdCen1*^*−/−*^ parasites was similar to one grown under laboratory conditions. The cGLP grade *LdCen1*^*−/−*^ parasites were found to be safe and non-toxic in hamsters and dogs even at 3 times the anticipated vaccine dose. When PBMCs from healed visceral leishmaniasis (VL) cases were infected with cGLP *LdCen1*^*−/−*^, there was a significant increase in the stimulation of cytokines that contribute to protective responses against VL. This effect, measured by multiplex ELISA, was greater than that observed in PBMCs from healthy individuals. These results suggest that cGLP grade *LdCen1*^*−/−*^ manufactured under cGMP complaint conditions can be suitable for future clinical trials.

## Introduction

Visceral leishmaniasis (VL)/kala-azar, caused by the protozoan parasite, either *Leishmania donovani* and *L. infantum* is a potentially fatal disease, with an estimated 50,000 to 90,000 cases worldwide annually as per leishmaniasis fact sheet 2021 by World Health Organization (https://www.who.int/news-room/fact-sheets/detail/leishmaniasis#:~:text=Visceral%20leishmaniasis%20(VL)%2C%20also,East%20Africa%20and%20in%20India). One of the major hurdles in VL elimination is the presence of asymptomatic cases as they can transmit the infection without getting noticed and the parasites may also persist for a longer period in the absence of treatment^1,2^. Owing to the limited drug options for VL, the development of resistance by *Leishmania* to most of the available drugs, and the prevalence of asymptomatic cases, vaccination seems to be the most viable option for the elimination of leishmaniasis^3–5^.

Induction of robust protective immunity against VL in the recovered patients, suggests that the development of a vaccine for VL is a feasible goal^6,7^. Vaccination could be an effective approach to reduce both symptomatic as well as asymptomatic cases and to achieve the VL eradication target of 2030^4,8,9^. Several vaccine candidates against leishmaniasis have been tried in the past, but no licensed vaccine is yet available for human use^6,10^.

One of the major concerns that limit the widespread use of live attenuated parasite vaccines is the absence of clear biomarkers related to their safety and protection^11^. Partially attenuated parasites can revert to virulent types and cause serious disease, especially in immunocompromised individuals. To rule out any such possibility of conversion, we developed a live attenuated *L. donovani* parasite by deleting both the alleles of the growth-regulating gene, centrin1 (*LdCen1*^*−/−*^)^12,13^. LdCen1 is a basal body-associated protein known to be involved in cell division^13^. *LdCen1*^*−/−*^ amastigotes (intracellular form) undergo continued karyokinesis without cytokinesis due to the absence of basal body duplication, resulting in the formation of large multinucleated cells followed by apoptosis^12^. This non-replicating parasite hence can be cultivated as promastigotes (extracellular form) in vitro. Extensive studies in animal models with *LdCen1*^*−/−*^ suggest, that they are safe, immunogenic, and protective against wild-type *L. donovani* infection, and also cross-protective against *L. major* that causes cutaneous leishmaniasis (CL) and *L. braziliensis* that causes mucocutaneous leishmaniasis^14–17^. Studies using human PBMCs also showed that they strongly promote protective pro-inflammatory cytokines in PBMCs of post-kala-azar-dermal leishmaniasis (PKDL) and healed VL (HVL) patients, who are already exposed to *Leishmania* infection^18^. These results suggest that *LdCen1*^*−/−*^ has great potential as a live attenuated vaccine candidate against leishmaniases. However, there are possibilities that a parasite grown at a large scale in the industry for the vaccine may behave differently compared to its small-scale culturing in the laboratory environment. Hence to confirm the attenuation and efficacy consistency of a large-scale cultivation of *LdCen1*^*−/−*^ in an industry setting, the cells were grown following current good laboratory practices (cGLP) at our industry partner Gennova Biopharmaceuticals, Pune, India, and the cells were used in subsequent toxicity studies. Here, we have presented the results of the detailed toxicity studies carried out in hamsters and dogs using cGLP grade of the parasite.

Recently, a detailed confirmation of protective immunity against the cGLP grade *L. major* centrin deleted live attenuated parasite as a vaccine candidate has been reported^19^. However, a preclinical toxicity analysis of a cGLP prepared live attenuated whole parasite as a vaccine before its use in clinical trials is needed. In this study we have outlined a comprehensive strategy of characterizing cGLP grade *LdCen1*^*−/−*^ parasites. We describe (1) preparation of consecutive batches of cGLP grade *LdCen1*^*−/−*^ parasites, including optimisation of batch cultivation steps, cell cryopreservation and stabilization/viability of frozen cells after thaw, parasites genetic stability, confirmation of its attenuation, (2) testing of toxicity of one of the batches and route of administration in animals, and (3) testing of the cGLP vaccine’s immunogenicity using the PBMCs from HVL patients and healthy controls. Here we present a detailed model of preclinical studies with the cGLP live attenuated vaccine parasitic cells, the needed approval processes to execute the studies, animal models used, ethical clearances obtained, number of sample sizes in the studies, replicas followed in the experiments, comprehensive toxicity analysis in the immunized animals, clinical samples used, and the data compared with the earlier results obtained using the laboratory strain of the vaccine with appropriate statistical validations. The presented protocol would help subsequently other researchers who would tend to validate preclinically their live parasite vaccines.

## Results

### cGLP *LdCen1*^*−/−*^ batch to batch consistency

#### Parasite cell growth and viability during fermentation

**Figure 1:**
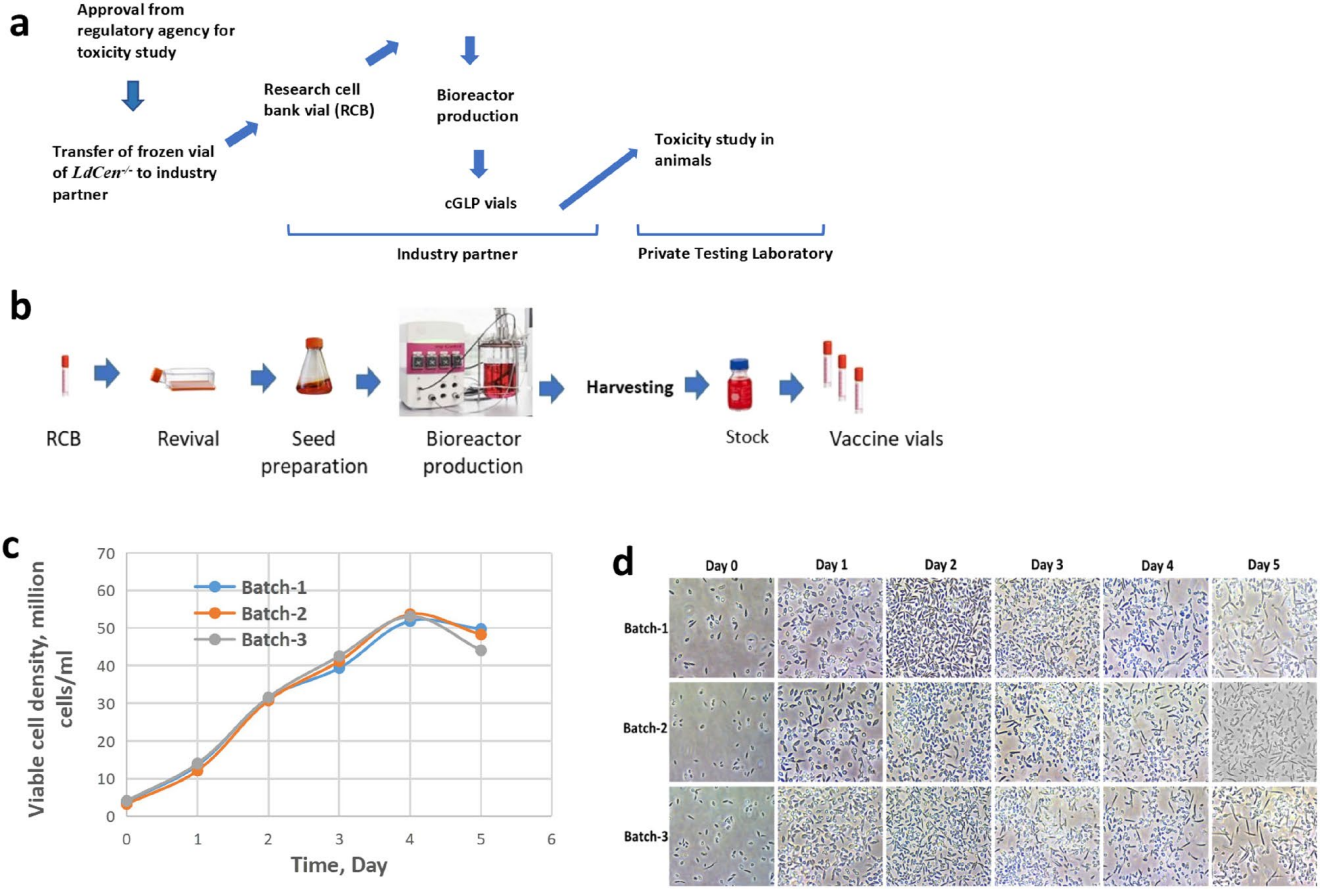
Preparation of cGLP cells of *LdCen1*^*−/−*^. (**a**) The scheme displays the entire protocol starting from the regulatory approval till the toxicity study in the animals through the industry generated cGLP of *LdCen1*^*−/−*^. (**b**) Diagrammatic representation of cGLP vials preparation by industrial partner. c. Growth profiles of three consecutive batches for cGLP cells counted by haemocytometer. d. Microscopic images (400X magnification) of bioreactor cultured cGLP cells on different days of growth.

The cGLP parasites were grown from the stored research cell bank of 2.5 × 10^6^ cells in 200 µL cryoprotectant prepared from the initial seed vial having similar number of stored cells (Fig. 1a). The preparation of the ready-to-use vials of cells through sequential volume escalation has been schematically described in Fig. 1b. The promastigote cells of *LdCen1*^*−/−*^ in the bioreactor culture displayed a sigmoid pattern of growth (Fig. 1c) and the cells’ density under microscope from day 1 to 5 are shown (Fig. 1d). As shown in Fig. 1c, *LdCen1*^*−/−*^ parasites reached their peak growth at about ~ 52 million cells per ml. All batches were harvested on day 5 when the culture reached a late stationary phase.

#### Parasite yield from the batch cultures

Each of the three batches of *LdCen1*^*−/−*^, yielded a mean cell density of 47 ± 3 × 10^6^ cells/ml in a 500 ml production batch in 5 days. The total parasite number from each batch was 23,700 ± 0.15 × 10^6^ cells (Table 1).

**Table 1:** Batch productivity.

Batch no.	Harvest cell density	Volume produced per batch	Total cells per batch
	× 10^6^ cells/ml	mL	× 10^6^ cells
Batch-1	49.8	500	24,900
Batch-2	48.3	500	24,150
Batch-3	44.1	500	22,050

#### Stability of the cGLP grade parasite

**Figure 2:**
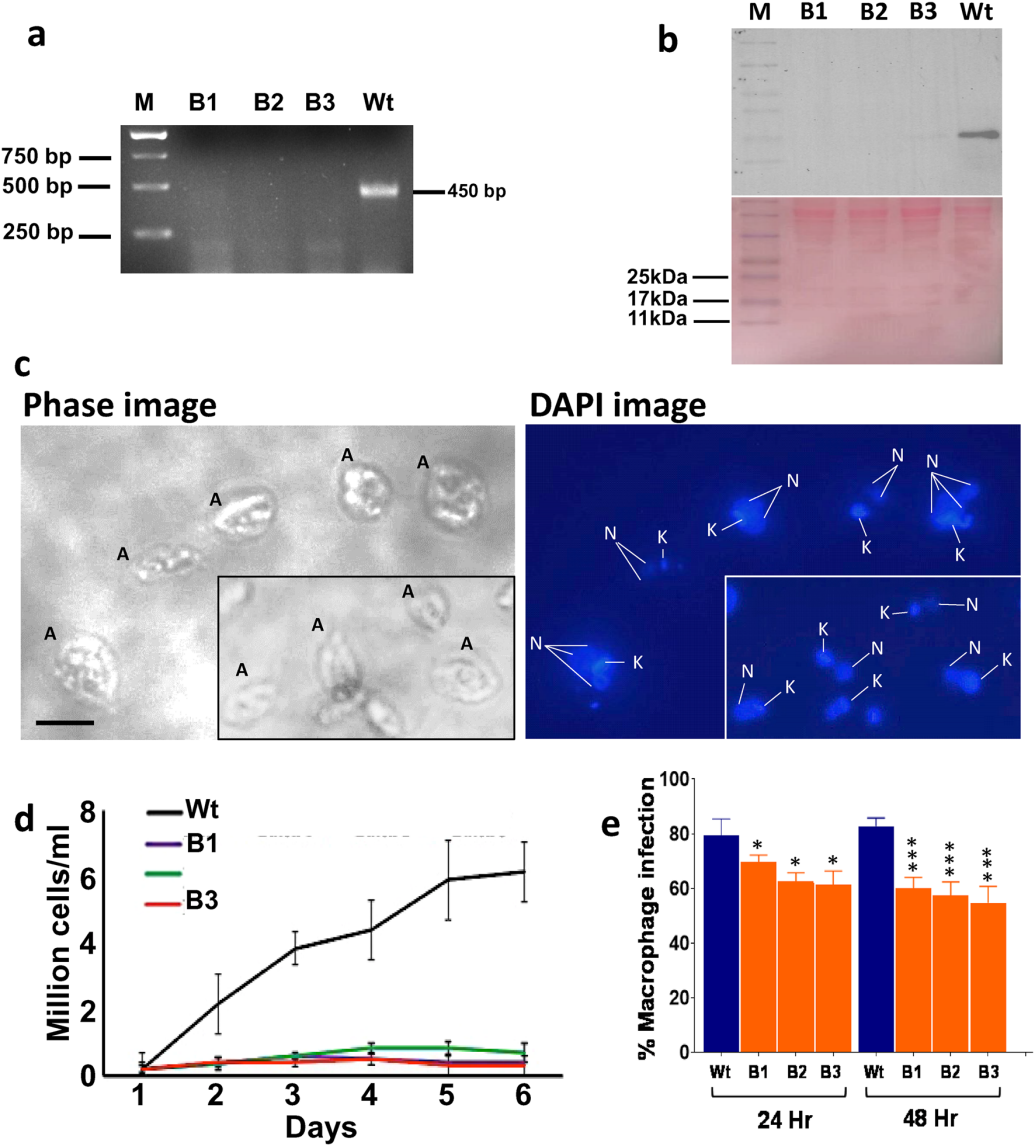
A Genetic confirmation and growth characteristics of cGLP amastigotes of the 3 batches. (**a**) PCR confirmation of the cGLP cells to know the cells were *LdCen1*^*−/−*^ comparing with the wild type cells (see the absence of amplification of centrin genes in the lanes from the DNA of cGLP of all the 3 batches (B1-B3). (**b**) Western blot analysis confirming of the cGLP cells to know the cells were *LdCen1*^*−/−*^ comparing with the wild type cells (see the absence of centrin1 protein in lanes B1-B3 of proteins of cGLP batches B1-B3; bottom panel shows the ponceau staining of the same gel as loadings of proteins). (**c**) Microscopic view of the batch 1 (B1) *LdCen1*^*−/−*^ cGLP cells to see the *LdCen1*^*−/−*^’s typical multinucleated (N) nature of amastigote (A) cells (with still single kinetoplast (K)) in them confirming the arrested cell division in those cells. Scale bar in all: 5 µM. (**d**) Growth of the three batches of the cGLP cells of *LdCen1*^*−/−*^ axenic amastigotes displaying the growth arrested nature of the *LdCen1*^*−/−*^ cells of all the batches as observed previously by us with the laboratory grade cells. (**e**) Percentage of infected human macrophages with the 3 batch parasites of *LdCen1*^*−/−*^ in vitro at various time points 48 h post-infection. T-test: *P < 0.05, ***P < 0.0005.

*A. Genetic Stability*: The genetic stability of the cGLP parasites in vitro was conducted to confirm the authenticity of the cells and make sure that they still resembled the original laboratory strain as reported^12^. The genomic DNA obtained from the cultured cells of cGLP from the three batches was subjected to PCR analysis using centrin gene-specific primers^12^ and confirmed the absence of the centrin1 gene in the cells of all the batches, confirming it to be *LdCen1*^*−/−*^ (Fig. 2a). Similarly, the total proteins obtained from the cultured cells of all cGLP batches were subjected to Western blot analysis using rabbit anti-centrin1 antibodies^12^ and confirmed the absence of centrin1 proteins in the cells (Fig. 2b).

In addition, the cells of the cGLP batches were sub-cultured into axenic amastigotes and confirmed their growth and cell structure under the microscope. The appearance of typical multinucleated cells of axenic amastigotes on day 2 in the culture due to arrest of the cell cycle in them compared to the control wild-type cells (having a typical single set of nucleus and kinetoplast; Fig. 2c) was observed (Fig. 2c)^12^. The growth study displays the growth-arrested nature of the *LdCen1*^*−/−*^ cells of all the batches (Fig. 2d). This confirms that *LdCen1*^*−/−*^ parasites grown under cGLP conditions behave like the lab-grown parasites^12^.

*b. Infectivity*: The infectivity and the associated safety and protective efficacy of *LdCen1*^*−/−*^ have been extensively studied ex vivo and in vivo in animal models as well as in clinically isolated human PBMCs, as described in our earlier publications^14–17,20,21^. To briefly confirm the infectivity of the cGLP cells in the human THP-1 cell line, we infected the cells with the cGLP promastigote cells in vitro*,* measured the percent of cells infected, and compared them with the infection due to the freeze–thaw wild-type cells. The results showed that initially (at 12 h), the infective nature of the cells was similar to that of wild-type cells. However, after 48 h, there was a significant reduction in the virulence of only the cGLP cells (Fig. 2e).

#### Viability of cGLP vaccine cells post cryopreservation

**Figure 3:**
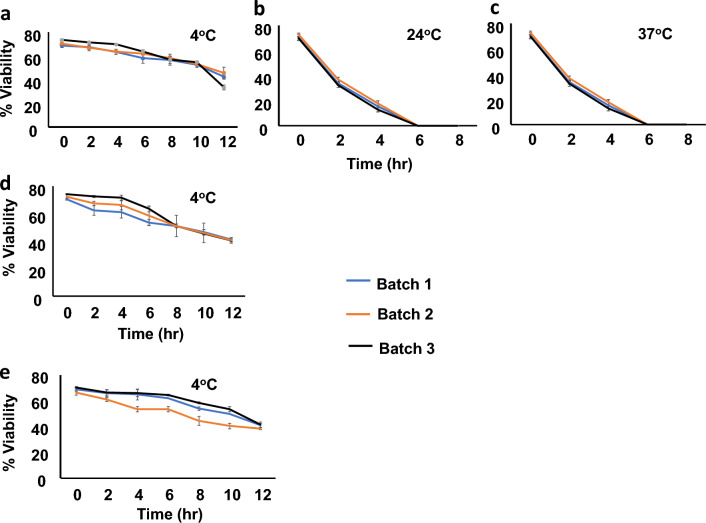
The viability test of three batches of cGLP cells (cryopreserved in 1X PBS of 5 million cells/100 µl) of *LdCen1*^*−/−*^ after thaw. (**a**–**c**) One month after cryopreservation; (**d**) Two months after cryopreservation; (**e**) Three months after cryopreservation. The temperature at which the maintenance of the cells is described in each graph (**a**–**e**).

The viability of 3 batches of cGLP *LdCen1*^*−/−*^ parasites was monitored over three months after cryopreservation. The results showed that the promastigote cells were ~ 70% viable after thawing and the viability decreased to ~ 50% when stored at 4 °C for 10 h (Fig. 3a, b and e). However, there was drastic decline in the viability of cells if stored at either 25 °C or 37 °C within 2 h post thaw (Fig. 3b and c) and the cells were all dead by 6 h (conducted with all the 3 batch cells). The cells were viable between 50 and 70% while kept at 4 °C in the refrigerator for 10 h uniformly with all the batch cells. Hence, we conclude that 4 °C could be the preferred temperature to store the parasites post-thaw until it is used for immunization. Depending on the viability of the thawed cGLP parasite cells stored at 4 °C, the conversion table in Supplementary Table 1 suggests the actual live cells (100%) as 1X and 3X doses to be used in the toxicity analysis in the animals. As there was little variability in cell viability between the 3 batches, batch ‘1’ cells were selected for use in the preclinical toxicity (PCT) analysis as per the cell number mentioned in the Supplementary Table 1.

#### Toxicity study in the animals

*A. Study 1: Single dose toxicity study of LdCen1*^*−/−*^* cGLP cells by subcutaneous (SC) and intradermal (ID) administration in hamsters*. This study was conducted to assess the safety and tolerability of the cGLP cells. These different routes of administration were attempted to choose an appropriate one for the subsequent studies. This study was also intended to provide information on important toxic effects, target organs and an estimation of the maximum tolerable dose (MTD). Both the administration studies were with 3 groups (G1: group 1 with placebo; G2: group 2 with 1 × vaccine dose and G3: group 3 with 3X vaccine dose), and each group had 6 hamsters (3 females and 3 males; Table 2; Supplementary Table 2). Parameters evaluated during the study period were survival, clinical signs, body weight, feed consumption, and necropsy findings.

**Table 2:** Method in brief of study/analyses of toxicity in animals due to cGLP *LdCen1*^*−/−*^.

Study name	Route of administration SC/ID & other details	Groups and sex (6 animals in each group)	Treatment	Study details
STUDY I Single dose toxicity study in Hamsters	SC Analysed on day 15	G1 (3♂ & 3♁)	G1: Placebo	Clinical observations: General and local examinations till 4 h after vaccination on Day 1 and once daily thereafter Body weights & Food consumption: On Day 0, 4, 8 and 15 Gross pathology: Day 15, gross lesions if any were collected
		G2 (3♂ & 3♁)	G2: 1X vaccine	
		G3 (3♂ & 3♁)	G3: 3X vaccine	
	ID Analysed on day 15	G4 (3♂ & 3♁)	G4: Placebo	
		G5 (3♂ & 3♁)	G5: 1X vaccine	
		G6 (3♂ & 3♁)	G6: 3X vaccine	
STUDY II Double dose toxicity study in Hamsters	SC First immunization on day 0. Analyse on day 30	G1 (3♂ & 3♁)	G1: Placebo	Clinical observations: General and local examinations till 4 h after vaccinations and once daily thereafter Body weights & Food consumption: weekly study Necropsy & Gross pathology: Day 30 and day 90, gross lesions if any were collected. Detailed pathology in individual organs including their weight
		G2 (3♂ & 3♁)	G2: 1X vaccine	
		G3 (3♂ & 3♁)	G3: 3X vaccine	
	SC Second immunization on day 30. Analyse on day 90	G4 (3♂ & 3♁)	G4: Placebo	
		G5 (3♂ & 3♁)	G5: 1X vaccine	
		G6 (3♂ & 3♁)	G6: 3X vaccine	
STUDY III Single dose toxicity study in dogs	SC Analysed on day 15	G1 (3♂ & 3♁)	G1: Placebo	Clinical observations: General and local examinations till 4 h after vaccination and once daily thereafter Body weights & Food consumption: On day 1, 4, 8 and 15 Rectal temperature: On day 1 and 2 Necropsy and Histopathology: day 15, All lesions if any were collected
		G2 (3♂ & 3♁)	G2: 1X vaccine	
		G3 (3♂ & 3♁)	G3: 3X vaccine	

**Figure 4:**
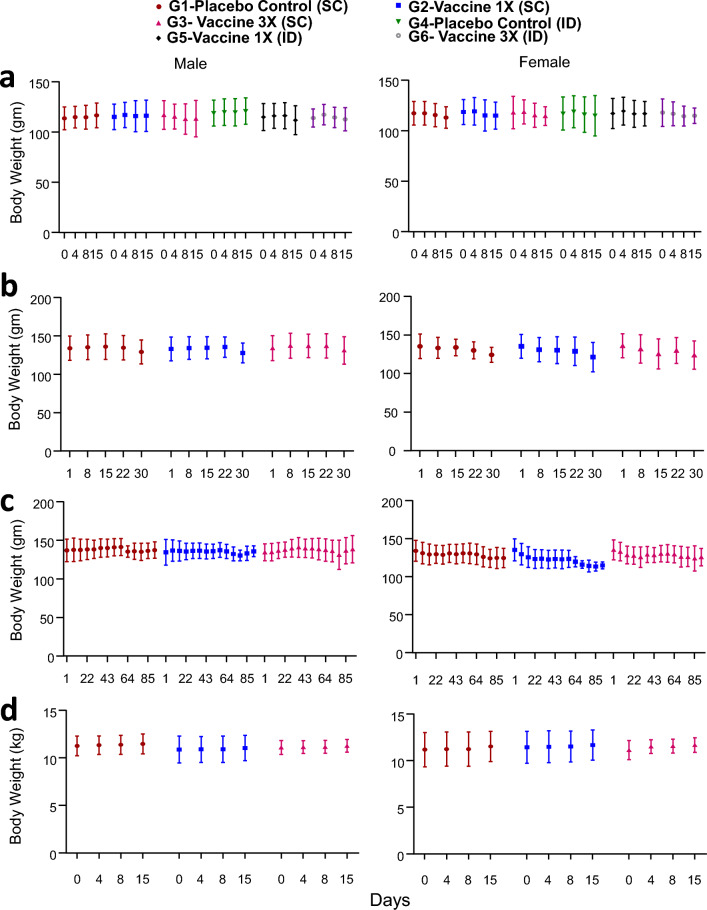
Summary graphs of average body weight of (**a**) hamsters from study I (15 days single dose study) after subcutaneous infection in G1–G3 groups; intradermal injections in G4–G6 groups; (**b** and **c**) hamsters from study II (with double dose) (on day-30 data of groups G1–G3 and on day-90 data of groups G4–G6) after subcutaneous infection; (**d**) dogs from study III (15 days single dose study) after subcutaneous infection in G1–G3 groups. In (**a**–**c**) each value is the average of 6 animals, whereas in ‘d’ it is the average of 3 animals. No statistically significant differences were found between experimental and placebo controls.

All animals were normal and were free from all visible clinical signs throughout the study period and survived up to scheduled termination. No treatment-related changes were observed in weekly body weight (Fig. 4a) and feed consumption (Supplementary Fig. 1A) compared to the control group. Neither any kind of lesion nor any disease-related clinical symptoms were noticed. The single SC and ID administration of cells in hamsters was found to be well tolerated without any clinical signs of toxicity. Based on these results, the MTD was found to be 9 million cells under the tested conditions. Comparing these two routes of injections, SC being less painful than ID^22^, we conducted the rest of the animal studies with SC administration.

*b. Study 2: Double dose immunization study in hamsters.* Animals from Groups G1–G3, that got one dose of vaccination (SC) on day1 were monitored till day 31, whereas the animals of groups G4–G6 with 2 doses of vaccines (administered subcutaneously on day 1 and day 31) were studied till day 91. In each case, there were 3 groups of animals (G1: group 1 with placebo; G2: group 2 with 1 × vaccine dose and G3: group 3 with 3X vaccine dose) and each group had 6 hamsters (3 females and 3 males; Table 2). Toxicity was assessed based on clinical signs, ophthalmoscopy, body weight, feed consumption, clinical and anatomic pathology. Animals were euthanized on Day 31 (G1–G3) and on Day 91 (G4–G6).

*Body weight, mortality and clinical signs*. There were no changes in the body weights or in any clinical signs of leishmaniasis/abnormalities attributable to treatment any of the placebo control and parasite-injected animals (Fig. 4b and c; Supplementary Table 3).

One female (T024/035) from G3 (3X dose treated) showed emaciation and dullness from Day 12 to Day 16 and was found dead on Day 17. Gross pathological examination revealed reduced thymus, blackish right eye, pus oozing out from cut open lacrimal gland, blackish enlargement in right lower jaw and abscess in right cerebrum. Mild suppurative inflammation in the brain and moderate infiltration of mononuclear cells in the liver and lungs were recorded on histopathological examination. These inflammatory findings might be the cause of death and were considered incidental and not attributed to treatment. One female (T024/046) from G4 (placebo control) had alopecia from Day 32 to Day 91, which was considered an incidental finding as there were no correlated changes in other parameters and the values were within the normal historical range.

*Detailed clinical observations*. All treated animals appeared normal without any clinical signs of toxicity during detailed veterinary examination (Supplementary Table 4), just as the control with the placebo. No musculoskeletal, nervous system, and respiratory disorders were noticed during the examination. The same female animal described above (T024/035) (from G3; 3X dose treated) from mid-dose exhibited incidental findings of dullness during week 2 and similarly, another female (T024/046) from high dose had alopecia from week 5 onwards.

*Ophthalmoscopic examination*. No ocular abnormalities were detected during the ophthalmoscopic examination carried out before treatment, on Day 30 (G1 and G3) and Day 91 (G4 and G6) (Supplementary Table 5).

**Figure 5:**
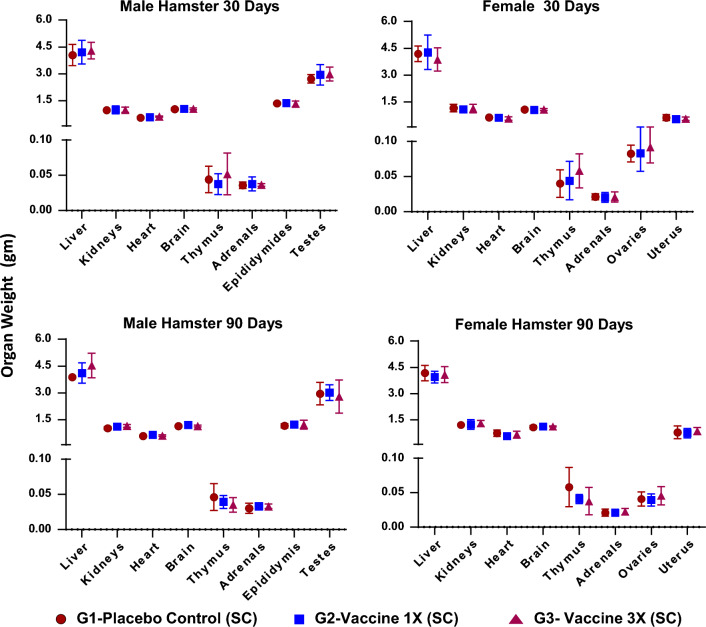
Summary graphs of average weight of organs of hamsters on day 30 (**a** and **b**) of groups G1–G3 and day 90 (**c** and **d**) of groups G4–G6. Each value is the average of 6 animals. No statistically significant differences were found between experimental and placebo controls.

*Body weight*. No statistically significant changes were observed in the mean body weight of test item-treated groups when compared with respective placebo control groups (Fig. 5).

*Feed consumption*. No treatment-related changes were observed in weekly feed consumption of all test item treated groups as compared to respective placebo control groups (Supplementary Fig. 1B; Supplementary Table 6). A statistically significant but marginal decrease in feed consumption was observed on Day 30 in G3 females when compared to G1 females compared to control. However, no associated changes were observed in body weight in the same group and no significant changes were observed in feed consumption in G6 females on Day 91. Further, an isolated significant increase in feed consumption was observed on Day 36 in G5 and G6 males when compared to G4 males. In the absence of changes in associated parameters or due to isolated occurrences, these findings were of no toxicological significance.

*Clinical pathology: Hematology*. No treatment-related adverse changes were observed up to the highest dose of 9 million cells/animal in any of the hematologic parameters on Day 31 and Day 91 as compared to respective placebo controls (Supplementary Table 7). Similarly, on day 91, no significant changes were observed in the hematology parameters tested (Supplementary Table 7).

*Clinical pathology: Coagulation parameters*. No significant immunization-related changes were observed in any of the coagulation parameters on Day 31 and Day 91 in parasite-injected animals as compared to respective placebo controls (Supplementary Table 8).

*Clinical chemistry*. No significant changes were observed in any of the clinical chemistry parameters on Day 31 and Day 91 in parasite-injected animals as compared to respective placebo controls (Supplementary Table 9). On Day 31, a statistically significant increase in globulin was observed in males treated with 3 million cells (G2). In the absence of a dose relationship, it was considered an incidental finding. Further, there was a decrease in creatinine in females (Day 31) and males (Day 91) treated with 9 million cells. The observed changes were minor or toxicologically insignificant.

*Clinical pathology: Urinalysis*. No treatment-related changes were observed in tested urine parameters (Supplementary Table 10). A statistically significant increase in mean urine pH was observed in males treated with 9 million cells (G6) when compared to Placebo control (G4) animals. Changes were considered incidental without a toxicological significance as there were no correlated changes in other parameters and values were within the normal historical range.

*Organ weight and relative organ weight*. No significant changes were observed in any of the absolute and relative organ weights on Days 31 and 91 as compared to respective placebo controls (Supplementary Tables 11–12; Fig. 5).

*Gross pathology*. No changes were observed in vaccinated animals sacrificed on days 31 and 91 (Supplementary Table 13) compared to control. On gross pathological examination of found dead female (T024/035) from G3 revealed a reduced thymus, blackish right eye, pus oozing out from cut open lacrimal gland, blackish enlargement in the right lower jaw and abscess in the right cerebrum. Unilateral adhesion of testes and epididymides with small seminal vesicle in one male (T024/025) and dark red colored enlarged ovary in one female (T024/033) were observed in 9 million cells dosed group on Day 31. Based on the histopathology evaluation of these organs, these findings were considered as incidental/spontaneous in nature and not attributed to test item treatment.

*Histopathology*. No histopathological findings were observed in 9 million cells dosed animals that were sacrificed on Day 91 (Supplementary Table 14). The histopathology findings observed in other organs were comparable between placebo control and 9 million cells dosed animals in terms of severity and incidences. Therefore, recorded histopathology findings were considered incidental/spontaneous and not attributed to test item treatment. Histopathological evaluation of femur bone with marrow revealed no abnormalities in vaccine-treated groups as compared to the vehicle control groups in both male and female hamsters. Further, a qualitative evaluation of the bone marrow smear did not reveal any abnormalities. No local reaction of erythema and edema was observed at the injection site in all vaccine-treated animals including vehicle control animals. Similarly, no pathological changes were identified during microscopic examination of the injection sites in all groups of both sexes. In conclusion, SC administration of *LdCen1*^*−/−*^ cGLP cells up to 9 million cells/animal was found to be well tolerated in hamsters up to 90 days, without any obvious signs of systemic toxicity under the study conditions.

a. Study 3: Toxicity analysis of single-dose *LdCen1*^*−/−*^ cGLP administered to dogs to assess the safety and tolerability of the parasite. This study was conducted to assess the safety and tolerability of cGLP cells in Beagle Dogs by single subcutaneous administrations. The results shown here are on day 15 post-immunization. The SC administration was with 3 groups (G1: group 1 with placebo; G2: group 2 with 1 × vaccine dose and G3: group 3 with 3X vaccine dose) and each group was with 6 dogs (3 females and 3 males; Table 2). Toxicity was assessed based on clinical signs, body weight, food consumption, clinical, Electrocardiogram (ECG) recording and anatomic pathology. Animals were sacrificed on Day 15. No treatment-related mortality and clinical signs were observed till day 15 of the study period up to the highest dose of 9 million cells/animal in both male and female dogs. No abnormal changes were observed during the detailed clinical examination. Body weight and food consumption in all the groups were comparable to the respective placebo control groups throughout the experimental period in both sexes (Fig. 4d). No treatment-related changes were observed in any of the electrocardiogram parameters evaluated in animals treated at 3 million, and 9 million cells/animal as compared to treatment with the placebo control (Supplementary Table 15). No changes attributable to treatment were observed in any of the hematology, coagulation and clinical chemistry parameters tested as compared to placebo control (Supplementary Tables 16–18). Gross pathological observations of all animals did not reveal any abnormalities. The single SC administration of cGLP cells in Beagle Dogs was found to be well tolerated without any clinical signs of toxicity. Based on these results, the MTD of was found to be 9 million cells/animal under tested conditions.

#### Measurement of immunoprotection of cGLP ***LdCen1***^***−/−***^ in human PBMCs

**Figure 6:**
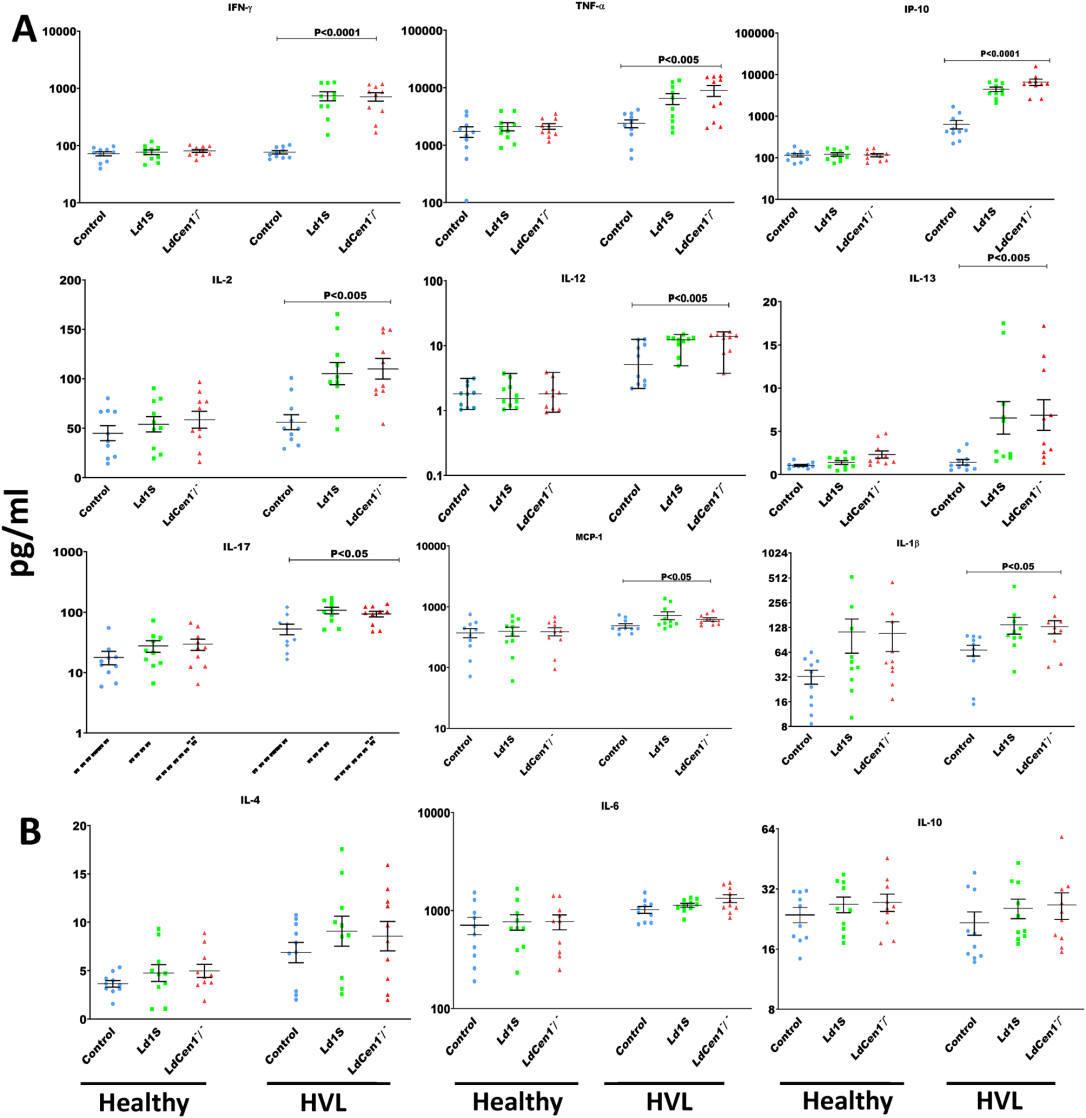
In the supernatants of PBMCs from Healthy (n = 10) and HVL (n = 10) individuals in response to wild type (Ld1S) and cGLP *LdCen1*^*−/−*^ parasite, level of protective cytokines (**a** panel) against leishmaniasis progression (IL-2, IL-12, IL-13, IFN-γ, TNF-α, IL-10, IL-17, MCP-1 and IL-1β) and the level of cytokines in increasing the susceptibility (**b** panel) towards leishmaniasis progression (IL-4, IL-6 and IL-10). In all the graphs Ld1S represents wild-type cells and *LdCen*^*−/−*^ denotes the cGLP cells. Data are given in Mean ± SEM (pg/ml). Significance was determined by Mann–Whitney U test. *P* < 0.05 is considered statistically significant.

*A. Cytokines involved in promoting host protection against leishmaniasis were induced by cGLP LdCen1*^*−/−*^* in HVL individuals.* Cytokines (IL-2, IL-12, IL-13, IFN-γ, TNF-α, IP-10, IL-17, MCP-1 and IL-1β) that play an important role in protecting leishmaniasis (Th1 response), were measured in PBMC supernatants. It was observed that there was a significant increase in the release of all studied cytokines in PBMC supernatants of HVL after infection with both wild-type as well as cGLP *LdCen1*^*−/−*^ parasites in comparison to the control uninfected PBMCs (Fig. 6A). In the healthy group, except IL-13, no significant difference in the production of any other cytokine was found in PBMCs after infection with Ld1S and *LdCen1*^*−/−*^ in comparison to control of uninfected cells**.** Further, this observed production of IL-13 in the healthy group was only observed in the cGLP *LdCen1*^*−/−*^ parasite-infected PBMCs. In HVL, secretion of IL-13 was observed with both wild-type and cGLP parasites infected PBMCs and the level of significance (*P* < 0.005) was more compared to the healthy group (*P* < 0.05). Moreover, the level of production of all cytokines obtained with the cGLP parasites in both study groups was similar to that with the wild-type parasites.

*b. Cytokines involved in increasing susceptibility to leishmaniasis were not induced by cGLP LdCen1*^*−/−*^* in both the studied groups*. Under this study, 3 cytokines (IL-4, IL-6 and IL-10), with a role in increasing susceptibility towards leishmaniasis progression (Th2 response), were estimated in PBMC supernatants. No significant production of any cytokine was observed after infection with wild-type or cGLP *LdCen1*^*−/−*^ parasites in comparison to the control uninfected PBMCs in both the groups examined i.e., healthy and HVL (Fig. 6B).

*c. Granzyme B is stimulated by cGLP LdCen1*^*−/−*^ Granzyme B, a serine protease, is mainly secreted by cytotoxic T-Cells (CTLs) and Natural killer (NK) cells. It is a potent inducer of apoptosis in target cells. Granzyme B level was determined in the cell supernatant of PBMCs of healthy and HVL (n = 6) infected with wild-type and cGLP *LdCen1*^*−/−*^ parasites. An increase in the release of Granzyme B was only observed in blood PBMC supernatants of HVL group infected with wild-type (Mean ± SEM = 381.9 ± 163.2) and *LdCen1*^*−/−*^ (Mean ± SEM = 404.2 ± 179.0 pg/ml) parasites as compared to the control uninfected cells (Mean ± SEM = 126.1 ± 50.98), not with the healthy group. The level of the release induced by *LdCen1*^*−/−*^ was comparable to that of the wild-type (Supplementary Fig. 2). However, this observed difference in the release of the infected cells compared to the control of uninfected cells was not significant (*P* > 0.05), which might be due to small sample size.

## Discussion

Detailed studies on the animal safety and immunity in a gene deleted (*LdCen1*^*−/−*^) live attenuated *L. donovani* vaccine candidate have been described through several of our earlier studies and in a recent review^4^. In addition, a pre-clinical evaluation of the safety and immunogenicity of cGLP grade *L. major* centrin^−/−^ parasites against sand fly infection was recently demonstrated^19,23^. However, a detailed study describing the preclinical toxicity evaluation of cGLP grade of such live attenuated parasites as a vaccine in the animals/human cells starting from the optimization of the culture of the industrial grade parasite in the bioreactors, freeze transporting followed by viability and genetic stability assessment and evaluations experimentally in animals and human cells using freeze thawed cells (cGLP) is still needed. The current study addressed these aspects for the first time and an optimized protocol and data are presented here.

The development of a safe and effective vaccine against the life-threatening infectious disease, VL/kala-azar is a long-term goal. Towards this goal, we have developed a replication-deficient live attenuated *L. donovani* parasite, which has shown good potential as a vaccine candidate in studies in the human PBMCs^14,16–18^ and animal models^21,24^. The present study was carried out using cGLP grade *LdCen1*^*−/−*^ parasite grown in the industrial setting to ensure that the results obtained are consistent with the laboratory grade parasite, to serve as a prerequisite test for the clinical studies. In addition, the report also carries the detailed toxicity study carried out in the animals for the first time in consultation with the regulatory agencies, as there is no precedence of such a preclinical procedure available to test whole live parasites as a vaccine.

Genetic recombination and introgression, contributing to the emergence of new parasite species could be a possibility in nature^25^. However, since 2009, with several reports on the stability of our gene-deleted parasite in animal models, we found genetic stability in the mutant parasite, without a sign of reverting to virulent form^12,14,24^. Moreover, in our live attenuated parasite, the mutation is irreversible, since the deletion is of both the alleles of the gene, centrin1 in *L. donovani,* whose homologous sequence is not present in higher eukaryotes including humans.

A controlled culture condition in the bioreactor, optimizations described for this study rendered a consistency between lot-to-lot preparation, which is mandatory for the regulating agencies to approve clinical studies. The consistency of parasite character among different lots was observed for cell number yield, genetic and cell attenuation in vitro in the macrophages. The study also revealed the viability of the freeze–thaw parasites of different lots (prepared at different times) uniformly showing reduced viability from initially 70% reducing to ~ 50% by 10 days. This will help to assess the number of cells to be used as a single vaccine dose within 10 h after transport to vaccination into remote places, where deep freezers are not easy to access. A study reported close to 90% viability after cryopreservation for just 20 min at − 80 °C yields with the wild-type *L. amazonensis*^26^. Whereas in our case the parasites were weakened by gene deletion that grows slightly slow even as promastigotes in culture^12^. Such cells of cGLP grade were cryopreserved at − 80 °C duly following the recommended procedures of slow cooling to − 80 °C (plus fast thawing at the time of use)^26^, stored for several months at the partner industry and transported in dry ice box having thermo-monitors to our university and upon receipt, they were again stored at − 80 °C for further use on a different date. Hence to the best of our knowledge, ours is the first such report describing the cold chain transport and viability counting of the cryopreserved cGLP of live attenuated *Leishmania* parasite.

Studies suggest that recovery from VL infection is favored by the induction of cell-mediated immunity followed by the production of pro-inflammatory cytokines by Th1 cells, while its progression is associated with the production of anti-inflammatory cytokines by Th2 cells^27^. Hence, an ideal vaccine candidate of VL should be capable of stimulation of Th1 cell-mediated pro-inflammatory cytokine response. In the present study, significant stimulation in the level of pro-inflammatory cytokines was observed in the PBMCs of HVL after infection with cGLP *LdCen1*^*−/−*^ parasites compared to the healthy human control; however, no such stimulation was found for anti-inflammatory cytokines. Further, the immune response induced by cGLP *LdCen1*^*−/−*^ parasites was comparable to that by wild-type parasites (Ld1S). These findings are consistent with the results obtained with laboratory-cultured *LdCen1*^*−/−*^ in our previous studies^18^. In addition to the previous observations, here we examined the stimulation of 4 more chemokines/cytokines i.e., IP-10, MCP-1, IL-1β, and IL-13 in the PBMCs, which are implicated in protecting against VL progression. All 4 of them were found to be stimulated in blood PBMCs of HVL after infection with cGLP *LdCen1*^*−/−*^ parasites.

Monocyte chemotactic protein-1 (MCP-1), a chemokine secreted in response to inflammatory signals, plays an important role in providing a protective immune response to the host by selective recruitment of neutrophils, monocytes, dendritic cells, and lymphocytes^28^. A previous study showed higher stimulation of MCP-1 in plasma samples of *Leishmania* antigen-stimulated whole blood collected from HVL and individuals living in the endemic area of *L. infantum*^29^*,* like the finding of the current study*.* Further, this study advocated MCP-1 as a very potent biomarker of cure and efficacy of drug treatment in VL and the selection and follow-up of the individuals in vaccine clinical trials. MCP-1 was also found to be a very robust biomarker in identifying asymptomatic infection^30^. IFN-γ-inducible protein-10 (IP-10) is produced by various cell types in response to IFN-γ mediated activation and it further promotes IFN-γ production. It also protects against VL progression in a manner like the MCP-1. Another study carried out by the same group found IP-10 as a robust marker of treatment response and cure from VL^29^.

IL-1β is a potent pro-inflammatory cytokine and helps in providing host resistance against *Leishmania* infection^31^. A study showed that there is a significantly higher level of IL-1β in serum samples of active VL patients as compared to the control uninfected individual^32^. Another study with antigen-stimulated PBMCs of CL patients found significant stimulation of IL-1β^33^. The role of IL-13 in leishmaniasis progression is conflicting. Some studies suggest that IL-13 contributes to increased susceptibility toward the cutaneous form of leishmaniasis^34^, while others suggest it plays a protective role^35^. However, VL studies suggest that it predominantly plays a protective role against disease progression. A study using mice infected with *L. donovani* as an experimental host of VL infection suggested that deletion of the IL-13 gene (IL-13^−/−^) resulted in decreased IFN-γ and increased IL-10 and IL-4 production, reduced granuloma maturation and drug responsiveness^36^, suggesting its pivotal role against VL progression. This report also similarly found an elevated level of IL-13 only in the HVL cases as a protective role.

We also measured the production of granzyme B in the PBMCs of HVL and healthy individuals after infection with cGLP *LdCen1*^*−/−*^ parasites. Granzyme B is a serine protease, produced by cytotoxic T cells and a potent inducer of apoptosis in the target cells^37^. Studies conducted in the CL suggest that granzyme B production is associated with protecting against disease progression^38^. The findings of the present study also indicate that there was an increase in granzyme B production in PBMCs of HVL cases after infection with cGLP *LdCen1*^*−/−*^ parasites. This further supports the potential of cGLP *LdCen1*^*−/−*^ as a live attenuated vaccine candidate.

Vaccination of asymptomatic VL cases is one of the finest approaches to control the spread of the disease. A study with a mice model of asymptomatic infection found that immunization with *LdCen1*^*−/−*^ generates multifunctional T-cell response and memory T-cell population and induces protection as evidenced by reduced wild-type parasite burden^20^. In addition, PBMCs, isolated from healthy volunteers from a non-endemic region, infected with centrin-deleted *L. major* parasite (*LmCen1*^*−/−*^) also stimulated enhanced IFN-γ compared to IL-10, which was consistent with the immunogenicity of this parasite in hamsters^19^. These data provide additional support that live attenuated parasites could be considered as prophylactic vaccines. To our knowledge ours is a novel preclinical study, where a vaccine candidate of cGLP grade has been comprehensively tested, meeting all the product characteristics. The cGLP grade *LmCen1*^*−/−*^ recently tested in hamsters and challenged with *L. donovani* and confirming cross-protection against the VL-causing parasite has also been demonstrated^19^ confirming cGLP grade cells in the in vivo studies in the animals demonstrating the utility of industrial grade cells for the vaccine efficacy evaluations.

## Conclusions

In conclusion, the present study describes a detailed procedure to monitor the genetic stability and toxicology aspects of the vaccine candidate including the needed regulatory approvals to similarly follow for other such eukaryotic whole live parasites as a vaccine. Based on the findings, the generated cGLP grade cells were genetically stable and the no-observed-adverse-effect level (NOAEL) of the parasites following SC administration in hamsters was 9 million cells/animal and demonstrated appropriate safety of vaccine at the proposed dose for humans. These findings further support the vaccine potential of cGLP *LdCen1*^*−/−*^. With the amount of data collected through this study determining the safety, and efficacy of the cGLP cells along with our earlier studies in various animal models using laboratory-grade parasites, the *LdCen1*^*−/−*^ cells as a vaccine can be tested clinically in humans or domestic dogs. Additionally, in this process, we described a detailed controlled preclinical study design to prepare, characterize, and evaluate the cGLP of *LdCen1*^*−/−*^ cells preclinically in ex vivo cells, toxicity analysis in the animals and protective immunity in the human cells.

## Materials and methods

### Preparation of cGLP grade parasites

Centrin1 gene deleted live growth attenuated *L. donovani* 1S (*LdCen1*^*−/−*^)^12^ and the parent *L. donova*ni 1S strain (MHOM/SD/62/1S) were used in all the experiments. Promastigotes and the axenic amastigotes of such strains were grown in vitro in T25 cm2 culture flasks (Corning, New York, US) in media as described earlier^12,39,40^. The cGLP grade cells were produced at 1L bioreactor scale having 500 ml of working volume. For the bioreactor production, each batch was inoculated with seed prepared by using T-flask and shake flask. In the seed development process, cells were revived from one frozen stock vial (having cell number 2.5 × 10^6^ cells stored in 200 µL cryoprotectant) and grown into a T-flask (25 cm^2^) containing 5 ml growth medium and incubated at 25 °C for the promastigote form^12,39,40^ (Fig. 1A-B). Further, cells were passaged after every second day in fresh growth medium to expand inoculum volume. Finally, 1L bioreactors having 500 ml of working volume were inoculated from the seed prepared in shake-flask. The initial cell density of the bioreactor was kept 2.0–4.0 × 10^6^ cells/ml. Bioreactor temperature and agitation were maintained at 25 °C and 100 rpm respectively throughout the run. The cells were measured using a hemacytometer and the viability of the cells was confirmed microscopically using trypan blue (Sigma Aldrich)^40^. The cells were harvested when they reached in the stationary phase after 5 days of cultivation (Fig. 1C, D). The harvested cells were pelleted and re-suspended in 1X PBS containing 8% glycerol (non-toxic cryoprotectant) and cell concentration of 60 × 10^6^ cells/ml to prepare a stock solution. From the prepared stock, the cells were aliquoted as 300 µl (6 million cells/100 µl) and stored at − 80 °C. Once the cells from the manufacturer under cGLP conditions were received by the testing facility (transported in dry ice), the cells were stored at − 80 °C. Such cells were used for the toxicity study in the animals.

Each parent and the batch cultures were tested/confirmed for their purity/absence of contaminants by periodically monitoring the cultures under the microscope and by plating an aliquot of cells on Luria Bertani (Sigma Aldrich) agar plates without any antibiotics and observing for contaminants if any following day. The parameters assessed for the quality confirmation of different lots of *LdCen1*^*−/−*^ promastigotes were: (a) enlarged cell structure and multinucleate of the axenic amastigotes in culture; (b) absence of centrin gene in the genome by PCR analysis compared with the DNA from the wild type parasites using primers reported previously and c) absence of *LdCen1* protein in the parasites in a Western blot analysis using anti *LdCen1* Ab^12^. These studies were conducted in the non-GLP condition in the laboratory.

### Optimization of cold-chain and management for vaccine delivery

After required periods of storage time, the cryotubes were removed from the freezer and thawed quickly at − 37 °C (water bath) and used directly in the preclinical studies. During the storage period, the cGLP vials were also transported from the industry to the testing sites by courier in the dry-ice boxes with temperature-controlled logistics (TCL) in place for real-time temperature monitoring during transport. Thus, right from the manufacturing till before its final thaw to use, the vaccine vials were monitored 24/7 for the maintenance of its stored temperature.

A detailed study of the viability of cells after thaw and its stability at different higher temperatures including room temperature was carried out for the *LdCen1*^*−/−*^ parasites. The promastigotes of *LdCen1*^*−/−*^ parasites stored at − 80 °C for different time periods were analyzed for the initial viability soon after thaw by measuring under a microscope with trypan blue vital stain and comparing with the similarly processed wild-type cells, wherever needed. In addition, the viability of the thawed cells was analyzed by extending its incubation at 4 °C and ambient temperature. This indicated the vaccine’s stability at a temperature feasible to maintain until it is used for vaccination.

### Survivability of cGLP *LdCen1*^***−/−***^ parasites in human macrophages

The macrophage cell line, THP-1 [Sigma Aldrich; monocyte culture maintained and differentiated to macrophages following exposure to lipopolysaccharides and lipoteichoic acid and used as per earlier report^41^ was infected with the three batches of the late stationary promastigotes of the cGLP *LdCen1*^*−/−*^ and the wild-type parasites as control. The parasites’ survival inside macrophages was measured by counting the infected macrophages and the internalized parasites by microscopy following published protocol^12^.

### Testing of cGLP product in animals for toxicity optimising different routes of administration

As per the regulatory norm, before testing the parasites in humans in phase I clinical study, it is mandatory to study the vaccine toxicity responses in animal models. Moreover, the toxicity analysis of *LdCen1*^*−/−*^ parasites in the animals was never carried out for the earlier laboratory grade of the parasite. Hence, the toxicity analysis in detail with the cGLP grade cryopreserved and thawed *LdCen1*^*−/−*^ parasites in hamsters and dogs was carried out at Vimta Laboratories, Hyderabad, India under the guidelines and approvals of the Institutional Biosafety Committee as well as the Review Committee on Genetic Manipulation, Department of Biotechnology, Ministry of Science and Technology, New Delhi, India. The studies were all conducted in compliance with The Organization of Economic Co-operation and Development (OECD) Principles of Good Laboratory Practice (GLP) for the testing of cells as specified by International [C (97) 186/Final] Legislation and in accordance with the approved study plan and following all relevant SOPs. The type of animals, their groups in the study, route of vaccine administration, duration of incubation and the type of analysis at the end of experiments are described briefly in Table 2 and detailed in Supplementary Table 2.

Detailed animal care specific to hamsters and dogs, that includes environmental conditions, housing, diet and feeding and drinking water, has been shown in the Supplementary section: pages 8–9.

### Infectivity and Immune responses due to cGLP product in the human cells

We reported the elicitation of Th1 immune response of laboratory grown *LdCen1*^*−/−*^ parasites in the human PBMCs^18^. It is imperative to similarly confirm the infectivity and immune responses with the cGLP product, in the human PBMCs.

PBMCs isolation and stimulation**:** Blood PBMCs were isolated from healthy and healed VL individuals’ heparinized blood by density gradient centrifugation with Ficoll-Hypaque (Sigma-Aldrich, St Louis, MO) and cultured in RPMI medium as previously reported^18^. The PBMCs of 2 × 10^5^ were plated in 96 well flat bottom tissue culture plates and incubated with (i) only media (unstimulated control) (ii) phytohemagglutinin (PHA) (1 μg/mL) as a positive control and (iii) 1 × 10^4^ live wild-type *L. donovani* (LDS) or (iv) 1 × 10^4^ *LdCen1*^*−/−*^ parasites. After 120 h of incubation at 37 °C and 5% CO_2_, supernatants were collected and stored at − 70 °C until further analysis.

Cytokine levels in supernatants were determined using the Human Cytokines, Bio-PlexPro™ (BIO-RAD) kit according to the manufacturer's protocol. Th1/Th2/Th17 cytokines were analyzed as reported previously^18^. The concentration of cytokines was calculated using software provided by the manufacturer (Bio-Plex Manager Software).

Parasite survival in macrophages: Macrophage cell line, THP-1 was infected with the cGLP parasite and macrophage ratio (10:1) and the wild-type parasites as control as late stationary promastigotes and the survival of the parasite inside macrophages were measured by counting the infected macrophages and internalized parasites by microscopy by following published protocol^12^.

### Human study populations and ethical consideration

The study was conducted in two groups: healthy (n = 10) and healed VL (n = 10). The sample size was determined based on the previous immunogenicity studies in humans^18^ using the published method^42^. People coming from VL non-endemic areas and negative for rk39 strip test (antigen test to diagnose VL)^43^ were included in the healthy group, while those having previous history of VL and treated at least 1 year before recruitment in the study were included in healed VL category. The study for the recruitment of both healthy individuals and healed VL groups was approved and carried out under the guidelines of the Ethical Committee of the Vardhman Mahavir Medical College and Safdarjung Hospital, New Delhi, India, and the samples were collected at the Department of Dermatology, Safdarjung Hospital. Informed consent was obtained from all the individuals recruited in the study.

### Multiplex cytokine and Granzyme B ELISA

Multiplex cytokine ELISA was performed with 50 µl of collected PBMC supernatants using Human Cytokines, Bio-PlexPro™ (Bio-Rad) kit as per the manufacturer protocol. A total of 12 cytokines, involved in providing host protection (IFN-γ, TNF-α, IP-10, IL-2, IL-12, IL-13, IL-17, MCP-1 and IL-1β) and increasing disease susceptibility (IL-4, IL-6 and IL-10) were studied. Reading was taken on a multiplex array reader instrument from Luminex™ Instrumentation System (Bio-Plex Workstation from Bio-Rad Laboratories) and concentrations were calculated using software provided by the manufacturer (Bio-Plex Manager Software). Granzyme B ELISA was also performed in 50 µl of PBMC supernatants using Human Granzyme B ELISA kit (Thermo Fisher Scientific) as per the manufacturer’s protocol.

### Statistical analysis

Data obtained in multiplex cytokine ELISA and Granzyme B ELISA were analyzed using Graph Pad Prism software (version 5.0). Results were represented as Mean Mean ± Standard Error Mean (SEM) in pg/ml. *P* value < 0.05 was considered statistically significant. Significance was determined by Mann–Whitney U test. Whereas the statistical analysis for the significance for the body/organ weight was measured by 2way ANOVA (Tukey’s multiple comparisons test).

### Ethical Clearances/statements

Various ethical clearances necessary to execute the studies were obtained by the respective institutes and industries. Animal Ethics Clearance was obtained by Vimta Laboratories, Hyderabad, AP, India (#VLS/IBSC-01/2017); Preclinical toxicity in the animals was approved by the review committee on genetic manipulation, Department of Biotechnology, (RCGM) (BT/BS/17/135/2004 = PID), New Delhi, India. Institutional Ethics Committee approval was obtained by National Institute of Pathology (IEC-NIP 2/3/17/03), New Delhi, India. Institute Biosafety Committee approval was obtained by the Department of Molecular Medicine (JH/DBT/FS/IBSC-03/2017), Jamia Hamdard, New Delhi, India. In addition, RCGM approved separately for all the transfer of parasites from one institute to the other and to execute specific the studies described in this report (viz.BT/BS/17/374/2016-PID; BT/BS/17/99/2002-PID; BT/BS/17/135/2004-PID).

#### Animal ethics statement

We confirm that the study is reported following the approval of The Committee for the Purpose of Control and Supervision of Experiments on Animals (CPCSEA), India, and following the guidelines of the Association for the Assessment and Accreditation of Laboratory Animal Care International (AAALAC). As per the animal protocol followed, at the final time point of study before acquiring the tissues/organs, the animals were euthanized by CO_2_ asphyxiation followed by exsanguination.

### Supplementary Information

Supplementary Information.

## Data Availability

All data generated or analyzed during this study are included in this published article [and its supplementary information files].
