# Supplementary material for: Manufacturing and preclinical toxicity of GLP grade gene deleted attenuated Leishmania donovani parasite vaccine
Source: Sci Rep. 2024 Jun 25;14:14636. doi: 10.1038/s41598-024-64592-6 (PMC11199483; doi:10.1038/s41598-024-64592-6)
Supplement: Supplementary file 1 — Supplementary Information. [file 41598_2024_64592_MOESM1_ESM.docx]

**Methods**

Statistical analysis

The data from the toxicology studies were analysed using Graphpad Prism, version 4.00. Statistical comparisons were carried out for all continuous data between treatment and control groups using parametric (one-way analysis of variance (ANOVA), Dunnett's t-Test) or non-parametric (Kruskal-Wallis test, Mann Whitney’s Test) test procedures. The choice of parametric or non-parametric test was based on whether the groups satisfy the homogeneity of variance as evaluated by Bartlett’s test. Statistical significance was evaluated at p≤0.05 and/or p≤0.01. All quantitative data was summarized and expressed as Mean ± SD. Comparison between two groups were done by Student’s t-test.

**Supplementary Data**

***Supplementary Figure 1: Summary graphs of feed consumption (g) of hamsters (A) in study 1: SC-Subcutaneous (groups G1-G3), ID-Intradermal (groups G4-G6); (B) in study 2 by subcutaneous administration of vaccine in hamsters with single dose study till 15 days (groups G1-G3) and with double dose study till 90 days (groups G4-G6) *- = Significant at p≤0.05 as compared to the respective placebo controls. No statistically significant differences were found between experimental and placebo controls.; Placebo ‘*’.***

For the study 1 (A) there were 6 groups; 3 groups for SC route: Placebo Control, Vaccine (1X), Vaccine (3X), 3 groups for ID route: Placebo Control, Vaccine (1X), Vaccine (3X). Each group had 12 hamsters (6 females and 6 males). See Supplementary Table 1.

For the study 2 (B) 6 groups- 3 groups (G1, G2, G3) with single dose - placebo control, Vaccine (1X), Vaccine (10X), 3 groups (G4, G5, G6) with two doses (G4, G5, G6) placebo control, vaccine (1X), Vaccine (3X). Each group had 12 hamsters; Males 54; Females 54 (18 males + 18 females were sacrificed on day 31 and day 91 (G1, G2, G3), 18 males + 18 females to be sacrificed on day 91 (G4, G5, G6). See Supplementary Table 1.


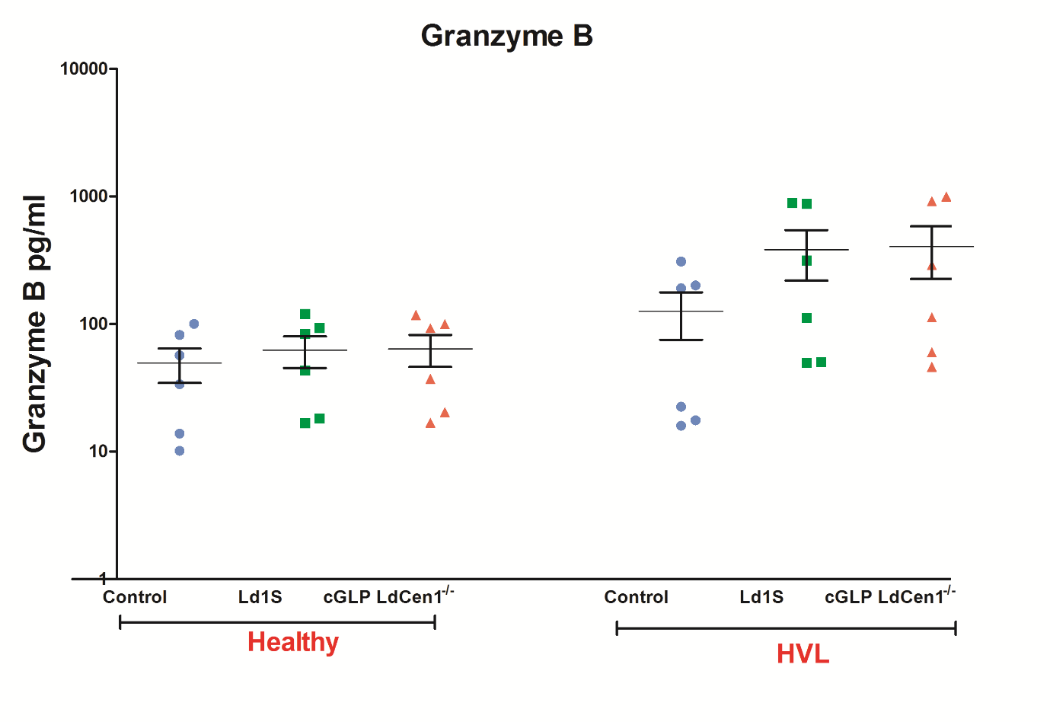


***Supplementary Figure 2: Level of Granzyme B in supernatant of peripheral blood mononuclear cells (PBMCs) from Healthy (n=6) and HVL (n=6) individuals in response to wild type (Ld1S) and cGLP LdCen1^-/-^ parasite. Data are given in Mean ± SEM (pg/ml). Significance was determined by Mann-Whitney U test. P < 0.05 is considered statistically significant.***

**Supplementary Table 1: Conversion for actual live cells as 1X and 3X doses from BATCH 1 based on the cell viability after thawing and storing at 4^o^C over a period of time.**

| Hours | % viable cells | No of cells as 1X  (Millions) | No of cells as 3X  (Millions) |
| --- | --- | --- | --- |
| 0 | 74 | 4.1 | 12.3 |
| 2 | 72.5 | 4.1 | 12.3 |
| 4 | 71.5 | 4.2 | 12.6 |
| 6 | 63.5 | 4.7 | 14.1 |
| 8 | 51 | 5.9 | 17.7 |
| 10 | 45.5 | 6.6 | 19.8 |

**Supplementary Table 2: Detailed method of study/analyses of toxicity in animals due to cGLP *LdCen1^-/-^***

| Study 1. Single dose toxicity (acute) study of cGLP grade vaccine in hamster by subcutaneous (SC) and intradermal (ID) administration | |
| --- | --- |
| **Objective** | To assess safety of *LdCen1^-/-^* vaccine after single dose administration in hamsters |
| **Route of administration** | Subcutaneous or intradermal |
| **Groups** | 6 groups  3 groups for SC route: Placebo Control, Vaccine (1X), Vaccine (3X), 3 groups for ID route: Placebo Control, Vaccine (1X), Vaccine (3X) |
| **Sex & number** | Males 36; Females 36 |
| **Dosing schedule** | Single Dose on Day 1 |
| **Clinical observations** | General and local examinations till 4 hours after vaccination on Day 1 and once daily thereafter |
| **Body weights** | On Day 1, 4, 8 and 15 |
| **Necropsy** | Day 15, gross lesions if any were collected |
| **Histopathology** | All gross lesions |
|  | |
| **Study 2. Double dose toxicity (sub-chronic) study of cGLP grade vaccine in hamster by subcutaneous administration** | |
| **Objective** | To assess safety of *LdCen1^-/-^* vaccine by SC administration in hamsters |
| **Route of administration** | Subcutaneous |
| **Groups** | 6 groups- 3 groups (G1, G2, G3) with single dose - placebo control, Vaccine (1X), Vaccine (10X), 3 groups (G4, G5, G6) with two doses (G4, G5, G6) placebo control, vaccine (1X), Vaccine (3X) |
| **Sex & number** | Males 54; Females 54 (18 males + 18 females were sacrificed on day 31 and day 91 (G1, G2, G3), 18 males + 18 females to be sacrificed on day 91 (G4, G5, G6) |
| **Dosing schedule** | Single dose on day 1 for groups G1, G2, G3, two doses: one on day1 and other on day 31 for groups G4, G5, G6 |
| **Clinical observations** | General and local examinations till 4 hours after vaccination and once daily thereafter |
| **Body weights** | Weekly |
| **Feed consumption** | Weekly |
| **s** | Hematology, clinical chemistry and immunology: on day31(G1, G2, G3), and 91 for all 6 groups (6 animals/sex/ group). Hematology – red blood cells, Hemoglobin, hematocrit, mean corpuscular volume, mean corpuscular hemoglobin, Mean corpuscular hemoglobin concentration, platelet, white blood cell, Differential Leukocyte Count, Reticulocyte count.  Clinical chemistry-Creatinine, blood. Urea nitrogen, glucose, total protein, albumin, globulin, albumin: globulin ratio, total bilirubin, aspartate aminotransferase, alanine aminotransferase, alkaline phosphatase, triglycerides, cholesterol  Immunology: Pro and anti-inflammatory cytokines |
| **Necropsy** | Interim sacrifice on day 31 and terminal sacrifice on day 91. Full list of tissues to be analyzed is mentioned below upon gross lesions if any:  adrenal glands, aorta, bone (femur) and articulation, bone (sternum) with bone marrow, brain, bronchi (mainstem), caecum, colon, duodenum, epididymites, eyes, heart, ileum, injection site(s) (a sample to be taken from the area injected), jejunum, kidneys and ureters, larynx, liver, lungs, lymph node (mandibular), lymph node (mesenteric), mammary gland, esophagus, optic nerves, ovaries and oviducts, pancreas, parathyroid glands, Peyer's patches, pituitary gland, prostate, rectum, salivary glands (mandibular, parotid, sublingual), sciatic nerves, seminal vesicles, skeletal muscle, skin, spinal cord (cervical, thoracic, lumbar), spleen, stomach, testes, thymus, thyroid glands, tongue, trachea, ureters, urinary bladder, uterus (horns + cervix), vagina |
| **Organ weights** | Organs to be measured were adrenal, brain, heart, kidney, liver, lung, spleen, testis / ovaries, uterus, epididymitis and thymus, (body to organ ratio to be calculated) |
| **Histopathology** | All gross lesions, adrenal glands, aorta, bone (femur) and articulation, bone (sternum) with bone marrow, brain, bronchi, caecum, colon, duodenum, epididymitis, eyes, heart, ileum, injection site(s) (a sample to be taken from the area injected), jejunum, kidneys and ureters, larynx, liver, lungs, lymph node (mandibular and mesenteric), mammary gland, esophagus, optic nerves, ovaries and oviducts, pancreas, parathyroid glands, Peyer's patches, pituitary gland, prostate, rectum, salivary glands, sciatic nerves, seminal vesicles, skeletal muscle, skin, spinal cord (cervical, thoracic, lumbar), spleen, stomach, testes, thymus, thyroid glands, tongue, trachea, ureters, urinary bladder, uterus (horns + cervix), vagina, all gross lesions |
|  | |
| **Study 3. Single dose toxicity study (acute) in beagle dogs** | |
| **Objective** | To assess safety of *LdCen1^-/-^* vaccine after single dose administration in dogs |
| **Route of administration** | Subcutaneous |
| **Groups** | 3 groups beagle dogs: control, Vaccine (1X), Vaccine (3X) |
| **Sex & number** | Males 9; Females 9 |
| **Dosing schedule** | Single dose on day 1 |
| **Clinical observations** | General and local examinations till 4 hours after vaccination and once daily thereafter |
| **Body weights** | On day 1, 4, 8 and 15 |
| **Rectal temperature** | On day 1 and 2 |
| **Necropsy** | day 15, gross lesions if any were collected |
| **Histopathology** | All gross lesions |

## **Animal Care (for hamsters)**

### Environmental Conditions

Animals were maintained under the following environmental conditions:

Temperature : 20.2 to 25.0 °C

Relative humidity : 49 - 70%

Light/dark cycle (photoperiod) : 12 h light & 12 h dark cycle

### Housing

Animals were housed individually in clean, autoclaved polysulfone cages (IVC; Length 425 mm x Breadth 266 mm x Height 185 mm) fitted with stainless steel grill tops having provision for holding pellet feed and water in bottles.

Cages and water bottles were changed twice a week. Autoclaved corn cob was used as the bedding material. Analysis of bedding material for specific microbiological load and chemical contaminants is carried out on a regular basis. Cage rotation was performed for every 15 days.

### Diet and Feeding

The animals were fed *ad libitum* with standard laboratory animal diet (SAFE Diets, France). Each batch of feed was analysed by the supplier for its nutritional components. Microbial and chemical contaminant analysis were carried out on a regular basis.

### Drinking Water

Fresh potable drinking water processed through a reverse osmosis system was provided *ad libitum* to animals in polysulfone bottles fitted with sipper tubes. Analysis of water for specific microbiological load and chemical contaminants is carried out on a routine basis.

The results of the bedding, feed and water analyses were retained along with the test facility records and a copy is maintained in the study raw data. No contaminants were present in the feed and water in sufficient quantities to affect the conduct or results of the study.

### Room Sanitation

Prior to occupancy, the experimental room was decontaminated. During the conduct of the study, the floor of the experimental room was cleaned daily. All worktops and floor were mopped with disinfectant solution on a day-to-day basis.

## **Animal Care (For DOGS)**

### Environmental Conditions

Animals were maintained under the following environmental conditions:

Temperature : 22.0 ± 23.4 °C

Relative humidity : 50 - 65%

Light/dark cycle (photoperiod) : 12 h light & 12 h dark cycle

Fresh air changes : Minimum of 12 fresh HEPA – filtered air-changes/hour

### Housing

Animals were kept in Room No.: Area 2. Animals were housed in stainless steel cages of size, approximately Length 4 feet x Breadth 3 feet x Height 5 feet. The animals were allowed for socializing in a specialized arena for at least one hour per day. Environmental enrichment was provided during the study.

### Diet and Feeding

The dogs were maintained on Standard dog chow - “Pedigree” manufactured by Mars International India Pvt. Ltd, New Delhi, India. The diet is pre-certified by the manufacturer for its nutritional components and maximum allowable concentrations of contaminants (e.g., heavy metals, aflatoxins, organophosphates, and chlorinated hydrocarbons). Further microbial and chemical contaminant analyses are carried out at test facility on a regular basis.

### Drinking Water

Fresh potable drinking water processed through a reverse osmosis system was provided *ad libitum* to all the dogs except for the duration of the dosing. Water samples were collected regularly and analysed for potential contaminants (specific microbiological load and chemical contaminants.

The results of the feed and water analyses were retained along with the test facility records and a copy is maintained in the study raw data. No contaminants were present in the feed and water in sufficient quantities to affect the conduct or results of the study.

### Room Sanitation

Prior to occupancy, the experimental area was thoroughly cleaned with a disinfectant. Pens were sanitized with disinfectants and cleaned daily during the study. All worktops and floor were mopped with disinfectant solution on a day-to-day basis.

**Supplementary Table 3: Summary of Clinical Signs in the repeated dose (90 days) subcutaneous toxicity study of *LdCen1^-/-^* in hamsters**

**Gender: Male No. of Animals/group/sex: 6**

| **Group & Dose** | **Clinical Signs** | **No. of Animals showing Clinical Signs** | | | | | | | | |
| --- | --- | --- | --- | --- | --- | --- | --- | --- | --- | --- |
|  |  | **Day** | | | | | | | | |
|  |  | **1** | | | **2** | **3** | **4** | **5** | **6** | **7** |
|  |  | **1h** | **2h** | **4h** |  |  |  |  |  |  |
| G1-Placebo Control | Normal | 6 | 6 | 6 | 6 | 6 | 6 | 6 | 6 | 6 |
| G2-Vaccine 1X | Normal | 6 | 6 | 6 | 6 | 6 | 6 | 6 | 6 | 6 |
| G3-Vaccine 3X | Normal | 6 | 6 | 6 | 6 | 6 | 6 | 6 | 6 | 6 |
| G4-Placebo Control | Normal | 6 | 6 | 6 | 6 | 6 | 6 | 6 | 6 | 6 |
| G5-Vaccine 1X | Normal | 6 | 6 | 6 | 6 | 6 | 6 | 6 | 6 | 6 |
| G6-Vaccine 3X | Normal | 6 | 6 | 6 | 6 | 6 | 6 | 6 | 6 | 6 |

| **Group & Dose** | **Clinical Signs** | **No. of Animals showing Clinical Signs** | | | | | | | |
| --- | --- | --- | --- | --- | --- | --- | --- | --- | --- |
|  |  | **Day** | | | | | | | |
|  |  | **8** | **9** | **10** | **11** | **12** | **13** | **14** | **15** |
| G1-Placebo Control | Normal | 6 | 6 | 6 | 6 | 6 | 6 | 6 | 6 |
| G2-Vaccine 1X | Normal | 6 | 6 | 6 | 6 | 6 | 6 | 6 | 6 |
| G3-Vaccine 3X | Normal | 6 | 6 | 6 | 6 | 6 | 6 | 6 | 6 |
| G4-Placebo Control | Normal | 6 | 6 | 6 | 6 | 6 | 6 | 6 | 6 |
| G5-Vaccine 1X | Normal | 6 | 6 | 6 | 6 | 6 | 6 | 6 | 6 |
| G6-Vaccine 3X | Normal | 6 | 6 | 6 | 6 | 6 | 6 | 6 | 6 |

| **Group & Dose** | **Clinical Signs** | **No. of Animals showing Clinical Signs** | | | | | | | |
| --- | --- | --- | --- | --- | --- | --- | --- | --- | --- |
|  |  | **Day** | | | | | | | |
|  |  | **16** | **17** | **18** | **19** | **20** | **21** | **22** | **23** |
| G1-Placebo Control | Normal | 6 | 6 | 6 | 6 | 6 | 6 | 6 | 6 |
| G2-Vaccine 1X | Normal | 6 | 6 | 6 | 6 | 6 | 6 | 6 | 6 |
| G3-Vaccine 3X | Normal | 6 | 6 | 6 | 6 | 6 | 6 | 6 | 6 |
| G4-Placebo Control | Normal | 6 | 6 | 6 | 6 | 6 | 6 | 6 | 6 |
| G5-Vaccine 1X | Normal | 6 | 6 | 6 | 6 | 6 | 6 | 6 | 6 |
| G6-Vaccine 3X | Normal | 6 | 6 | 6 | 6 | 6 | 6 | 6 | 6 |

**Supplementary Table 3: Continued**

**Gender: Male No. of Animals/group/sex: 6**

| **Group & Dose** | **Clinical Signs** | **No. of Animals showing Clinical Signs** | | | | | | | |
| --- | --- | --- | --- | --- | --- | --- | --- | --- | --- |
|  |  | **Day** | | | | | | | |
|  |  | **24** | **25** | **26** | **27** | **28** | **29** | **30** | **31** |
| G1-Placebo Control | Normal | 6 | 6 | 6 | 6 | 6 | 6 | 6 | 6 |
| G2-Vaccine 1X | Normal | 6 | 6 | 6 | 6 | 6 | 6 | 6 | 6 |
| G3-Vaccine 3X | Normal | 6 | 6 | 6 | 6 | 6 | 6 | 6 | 6 |
| G4-Placebo Control | Normal | 6 | 6 | 6 | 6 | 6 | 6 | 6 | 6 |
| G5-Vaccine 1X | Normal | 6 | 6 | 6 | 6 | 6 | 6 | 6 | 6 |
| G6-Vaccine 3X | Normal | 6 | 6 | 6 | 6 | 6 | 6 | 6 | 6 |

| **Group & Dose** | **Clinical Signs** | **No. of Animals showing Clinical Signs** | | | | | | | | |
| --- | --- | --- | --- | --- | --- | --- | --- | --- | --- | --- |
|  |  | **Day** | | | | | | | | |
|  |  | **31** | | | **32** | **33** | **34** | **35** | **36** | **37** |
|  |  | **1h** | **2h** | **4h** |  |  |  |  |  |  |
| G4-Placebo Control | Normal | 6 | 6 | 6 | 6 | 6 | 6 | 6 | 6 | 6 |
| G5-Vaccine 1X | Normal | 6 | 6 | 6 | 6 | 6 | 6 | 6 | 6 | 6 |
| G6-Vaccine 3X | Normal | 6 | 6 | 6 | 6 | 6 | 6 | 6 | 6 | 6 |

| **Group & Dose** | **Clinical Signs** | **No. of Animals showing Clinical Signs** | | | | | | | |
| --- | --- | --- | --- | --- | --- | --- | --- | --- | --- |
|  |  | **Day** | | | | | | | |
|  |  | **38** | **39** | **40** | **41** | **42** | **43** | **44** | **45** |
| G4-Placebo Control | Normal | 6 | 6 | 6 | 6 | 6 | 6 | 6 | 6 |
| G5-Vaccine 1X | Normal | 6 | 6 | 6 | 6 | 6 | 6 | 6 | 6 |
| G6-Vaccine 3X | Normal | 6 | 6 | 6 | 6 | 6 | 6 | 6 | 6 |

**Supplementary Table 3: Continued**

**Gender: Male No. of Animals/group/sex: 6**

| **Group & Dose** | **Clinical Signs** | **No. of Animals showing Clinical Signs** | | | | | | | |
| --- | --- | --- | --- | --- | --- | --- | --- | --- | --- |
|  |  | **Day** | | | | | | | |
|  |  | **46** | **47** | **48** | **49** | **50** | **51** | **52** | **53** |
| G4-Placebo Control | Normal | 6 | 6 | 6 | 6 | 6 | 6 | 6 | 6 |
| G5-Vaccine 1X | Normal | 6 | 6 | 6 | 6 | 6 | 6 | 6 | 6 |
| G6-Vaccine 3X | Normal | 6 | 6 | 6 | 6 | 6 | 6 | 6 | 6 |

| **Group & Dose** | **Clinical Signs** | **No. of Animals showing Clinical Signs** | | | | | | | |
| --- | --- | --- | --- | --- | --- | --- | --- | --- | --- |
|  |  | **Day** | | | | | | | |
|  |  | **54** | **55** | **56** | **57** | **58** | **59** | **60** | **61** |
| G4-Placebo Control | Normal | 6 | 6 | 6 | 6 | 6 | 6 | 6 | 6 |
| G5-Vaccine 1X | Normal | 6 | 6 | 6 | 6 | 6 | 6 | 6 | 6 |
| G6-Vaccine 3X | Normal | 6 | 6 | 6 | 6 | 6 | 6 | 6 | 6 |

| **Group & Dose** | **Clinical Signs** | **No. of Animals showing Clinical Signs** | | | | | | | |
| --- | --- | --- | --- | --- | --- | --- | --- | --- | --- |
|  |  | **Day** | | | | | | | |
|  |  | **62** | **63** | **64** | **65** | **66** | **67** | **68** | **69** |
| G4-Placebo Control | Normal | 6 | 6 | 6 | 6 | 6 | 6 | 6 | 6 |
| G5-Vaccine 1X | Normal | 6 | 6 | 6 | 6 | 6 | 6 | 6 | 6 |
| G6-Vaccine 3X | Normal | 6 | 6 | 6 | 6 | 6 | 6 | 6 | 6 |

| **Group & Dose** | **Clinical Signs** | **No. of Animals showing Clinical Signs** | | | | | | | |
| --- | --- | --- | --- | --- | --- | --- | --- | --- | --- |
|  |  | **Day** | | | | | | | |
|  |  | **70** | **71** | **72** | **73** | **74** | **75** | **76** | **77** |
| G4-Placebo Control | Normal | 6 | 6 | 6 | 6 | 6 | 6 | 6 | 6 |
| G5-Vaccine 1X | Normal | 6 | 6 | 6 | 6 | 6 | 6 | 6 | 6 |
| G6-Vaccine 3X | Normal | 6 | 6 | 6 | 6 | 6 | 6 | 6 | 6 |

**Supplementary Table 3: Continued**

**Gender: Male No. of Animals/group/sex: 6**

| **Group & Dose** | **Clinical Signs** | **No. of Animals showing Clinical Signs** | | | | | | | |
| --- | --- | --- | --- | --- | --- | --- | --- | --- | --- |
|  |  | **Day** | | | | | | | |
|  |  | **78** | **79** | **80** | **81** | **82** | **83** | **84** | **85** |
| G4-Placebo Control | Normal | 6 | 6 | 6 | 6 | 6 | 6 | 6 | 6 |
| G5-Vaccine 1X | Normal | 6 | 6 | 6 | 6 | 6 | 6 | 6 | 6 |
| G6-Vaccine 3X | Normal | 6 | 6 | 6 | 6 | 6 | 6 | 6 | 6 |

| **Group & Dose** | **Clinical Signs** | **No. of Animals showing Clinical Signs** | | | | | |
| --- | --- | --- | --- | --- | --- | --- | --- |
|  |  | **Day** | | | | | |
|  |  | **86** | **87** | **88** | **89** | **90** | **91** |
| G4-Placebo Control | Normal | 6 | 6 | 6 | 6 | 6 | 6 |
| G5-Vaccine 1X | Normal | 6 | 6 | 6 | 6 | 6 | 6 |
| G6-Vaccine 3X | Normal | 6 | 6 | 6 | 6 | 6 | 6 |

Note: No reaction at the site of injection in all the groups throughout the study period.

**Supplementary Table 3: Continued**

**Gender: Female No. of Animals/group/sex: 6**

| **Group & Dose** | **Clinical Signs** | **No. of Animals showing Clinical Signs** | | | | | | | | |
| --- | --- | --- | --- | --- | --- | --- | --- | --- | --- | --- |
|  |  | **Day** | | | | | | | | |
|  |  | **1** | | | **2** | **3** | **4** | **5** | **6** | **7** |
|  |  | **1h** | **2h** | **4h** |  |  |  |  |  |  |
| G1-Placebo Control | Normal | 6 | 6 | 6 | 6 | 6 | 6 | 6 | 6 | 6 |
| G2-Vaccine 1X | Normal | 6 | 6 | 6 | 6 | 6 | 6 | 6 | 6 | 6 |
| G3-Vaccine 3X | Normal | 6 | 6 | 6 | 6 | 6 | 6 | 6 | 6 | 6 |
| G4-Placebo Control | Normal | 6 | 6 | 6 | 6 | 6 | 6 | 6 | 6 | 6 |
| G5-Vaccine 1X | Normal | 6 | 6 | 6 | 6 | 6 | 6 | 6 | 6 | 6 |
| G6-Vaccine 3X | Normal | 6 | 6 | 6 | 6 | 6 | 6 | 6 | 6 | 6 |

**Supplementary Table 3: Continued**

**Gender: Female No. of Animals/group/sex: 6**

| **Group & Dose** | **Clinical Signs** | **No. of Animals showing Clinical Signs** | | | | | | | |
| --- | --- | --- | --- | --- | --- | --- | --- | --- | --- |
|  |  | **Day** | | | | | | | |
|  |  | **8** | **9** | **10** | **11** | **12** | **13** | **14** | **15** |
| G1-Placebo Control | Normal | 6 | 6 | 6 | 6 | 6 | 6 | 6 | 6 |
| G2-Vaccine 1X | Normal | 6 | 6 | 6 | 6 | 6 | 6 | 6 | 6 |
| G3-Vaccine 3X | Normal | 6 | 6 | 6 | 6 | 5 | 5 | 5 | 5 |
|  | Emaciation  Dullness | - | - | - | - | 1 | 1 | 1 | 1 |
| G4-Placebo Control | Normal | 6 | 6 | 6 | 6 | 6 | 6 | 6 | 6 |
| G5-Vaccine 1X | Normal | 6 | 6 | 6 | 6 | 6 | 6 | 6 | 6 |
| G6-Vaccine 3X | Normal | 6 | 6 | 6 | 6 | 6 | 6 | 6 | 6 |

| **Group & Dose** | **Clinical Signs** | **No. of Animals showing Clinical Signs** | | | | | | | |
| --- | --- | --- | --- | --- | --- | --- | --- | --- | --- |
|  |  | **Day** | | | | | | | |
|  |  | **16** | **17** | **18** | **19** | **20** | **21** | **22** | **23** |
| G1-Placebo Control | Normal | 6 | 6 | 6 | 6 | 6 | 6 | 6 | 6 |
| G2-Vaccine 1X | Normal | 6 | 6 | 6 | 6 | 6 | 6 | 6 | 6 |
| G3-Vaccine 3X | Normal | 5 | 5 | 5 | 5 | 5 | 5 | 5 | 5 |
|  | Emaciation  Dullness | 1 | - | - | - | - | - | - | - |
|  | Found Dead | - | 1 | - | - | - | - | - | - |
| G4-Placebo Control | Normal | 6 | 6 | 6 | 6 | 6 | 6 | 6 | 6 |
| G5-Vaccine 1X | Normal | 6 | 6 | 6 | 6 | 6 | 6 | 6 | 6 |
| G6-Vaccine 3X | Normal | 6 | 6 | 6 | 6 | 6 | 6 | 6 | 6 |

**Supplementary Table 3: Continued**

**Gender: Female No. of Animals/group/sex: 6**

| **Group & Dose** | **Clinical Signs** | **No. of Animals showing Clinical Signs** | | | | | | | |
| --- | --- | --- | --- | --- | --- | --- | --- | --- | --- |
|  |  | **Day** | | | | | | | |
|  |  | **24** | **25** | **26** | **27** | **28** | **29** | **30** | **31** |
| G1-Placebo Control | Normal | 6 | 6 | 6 | 6 | 6 | 6 | 6 | 6 |
| G2-Vaccine 1X | Normal | 6 | 6 | 6 | 6 | 6 | 6 | 6 | 6 |
| G3-Vaccine 3X | Normal | 5 | 5 | 5 | 5 | 5 | 5 | 5 | 5 |
| G4-Placebo Control | Normal | 6 | 6 | 6 | 6 | 6 | 6 | 6 | 6 |
| G5-Vaccine 1X | Normal | 6 | 6 | 6 | 6 | 6 | 6 | 6 | 6 |
| G6-Vaccine 3X | Normal | 6 | 6 | 6 | 6 | 6 | 6 | 6 | 6 |

| **Group & Dose** | **Clinical Signs** | **No. of Animals showing Clinical Signs** | | | | | | | | |
| --- | --- | --- | --- | --- | --- | --- | --- | --- | --- | --- |
|  |  | **Day** | | | | | | | | |
|  |  | **31** | | | **32** | **33** | **34** | **35** | **36** | **37** |
|  |  | **1h** | **2h** | **4h** |  |  |  |  |  |  |
| G4-Placebo Control | Normal | 6 | 6 | 6 | 5 | 5 | 5 | 5 | 5 | 5 |
|  | Alopecia | - | - | - | 1 | 1 | 1 | 1 | 1 | 1 |
| G5-Vaccine 1X | Normal | 6 | 6 | 6 | 6 | 6 | 6 | 6 | 6 | 6 |
| G6-Vaccine 3X | Normal | 6 | 6 | 6 | 6 | 6 | 6 | 6 | 6 | 6 |

| **Group & Dose** | **Clinical Signs** | **No. of Animals showing Clinical Signs** | | | | | | | |
| --- | --- | --- | --- | --- | --- | --- | --- | --- | --- |
|  |  | **Day** | | | | | | | |
|  |  | **38** | **39** | **40** | **41** | **42** | **43** | **44** | **45** |
| G4-Placebo Control | Normal | 5 | 5 | 5 | 5 | 5 | 5 | 5 | 5 |
|  | Alopecia | 1 | 1 | 1 | 1 | 1 | 1 | 1 | 1 |
| G5-Vaccine 1X | Normal | 6 | 6 | 6 | 6 | 6 | 6 | 6 | 6 |
| G6-Vaccine 3X | Normal | 6 | 6 | 6 | 6 | 6 | 6 | 6 | 6 |

**Supplementary Table 3: Continued**

**Gender: Female No. of Animals/group/sex: 6**

| **Group & Dose** | **Clinical Signs** | **No. of Animals showing Clinical Signs** | | | | | | | |
| --- | --- | --- | --- | --- | --- | --- | --- | --- | --- |
|  |  | **Day** | | | | | | | |
|  |  | **46** | **47** | **48** | **49** | **50** | **51** | **52** | **53** |
| G4-Placebo Control | Normal | 5 | 5 | 5 | 5 | 5 | 5 | 5 | 5 |
|  | Alopecia | 1 | 1 | 1 | 1 | 1 | 1 | 1 | 1 |
| G5-Vaccine 1X | Normal | 6 | 6 | 6 | 6 | 6 | 6 | 6 | 6 |
| G6-Vaccine 3X | Normal | 6 | 6 | 6 | 6 | 6 | 6 | 6 | 6 |

| **Group & Dose** | **Clinical Signs** | **No. of Animals showing Clinical Signs** | | | | | | | |
| --- | --- | --- | --- | --- | --- | --- | --- | --- | --- |
|  |  | **Day** | | | | | | | |
|  |  | **54** | **55** | **56** | **57** | **58** | **59** | **60** | **61** |
| G4-Placebo Control | Normal | 5 | 5 | 5 | 5 | 5 | 5 | 5 | 5 |
|  | Alopecia | 1 | 1 | 1 | 1 | 1 | 1 | 1 | 1 |
| G5-Vaccine 1X | Normal | 6 | 6 | 6 | 6 | 6 | 6 | 6 | 6 |
| G6-Vaccine 3X | Normal | 6 | 6 | 6 | 6 | 6 | 6 | 6 | 6 |

| **Group & Dose** | **Clinical Signs** | **No. of Animals showing Clinical Signs** | | | | | | | |
| --- | --- | --- | --- | --- | --- | --- | --- | --- | --- |
|  |  | **Day** | | | | | | | |
|  |  | **62** | **63** | **64** | **65** | **66** | **67** | **68** | **69** |
| G4-Placebo Control | Normal | 5 | 5 | 5 | 5 | 5 | 5 | 5 | 5 |
|  | Alopecia | 1 | 1 | 1 | 1 | 1 | 1 | 1 | 1 |
| G5-Vaccine 1X | Normal | 6 | 6 | 6 | 6 | 6 | 6 | 6 | 6 |
| G6-Vaccine 3X | Normal | 6 | 6 | 6 | 6 | 6 | 6 | 6 | 6 |

| **Group & Dose** | **Clinical Signs** | **No. of Animals showing Clinical Signs** | | | | | | | |
| --- | --- | --- | --- | --- | --- | --- | --- | --- | --- |
|  |  | **Day** | | | | | | | |
|  |  | **70** | **71** | **72** | **73** | **74** | **75** | **76** | **77** |
| G4-Placebo Control | Normal | 5 | 5 | 5 | 5 | 6 | 6 | 6 | 6 |
|  | Alopecia | 1 | 1 | 1 | 1 | - | - | - | - |
| G5-Vaccine 1X | Normal | 6 | 6 | 6 | 6 | 6 | 6 | 6 | 6 |
| G6-Vaccine 3X | Normal | 6 | 6 | 6 | 6 | 6 | 6 | 6 | 6 |

**Supplementary Table 3: Continued**

**Gender: Female No. of Animals/group/sex: 6**

| **Group & Dose** | **Clinical Signs** | **No. of Animals showing Clinical Signs** | | | | | | | |
| --- | --- | --- | --- | --- | --- | --- | --- | --- | --- |
|  |  | **Day** | | | | | | | |
|  |  | **78** | **79** | **80** | **81** | **82** | **83** | **84** | **85** |
| G4-Placebo Control | Normal | 6 | 6 | 6 | 6 | 6 | 6 | 6 | 6 |
| G5-Vaccine 1X | Normal | 6 | 6 | 6 | 6 | 6 | 6 | 6 | 6 |
| G6-Vaccine 3X | Normal | 6 | 6 | 6 | 6 | 6 | 6 | 6 | 6 |

| **Group & Dose** | **Clinical Signs** | **No. of Animals showing Clinical Signs** | | | | | |
| --- | --- | --- | --- | --- | --- | --- | --- |
|  |  | **Day** | | | | | |
|  |  | **86** | **87** | **88** | **89** | **90** | **91** |
| G4-Placebo Control | Normal | 6 | 6 | 6 | 6 | 6 | 6 |
| G5-Vaccine 1X | Normal | 6 | 6 | 6 | 6 | 6 | 6 |
| G6-Vaccine 3X | Normal | 6 | 6 | 6 | 6 | 6 | 6 |

Note: No reaction at the site of injection in all the groups throughout the study period.

**Supplementary Table 4: Summary of Detailed Clinical Examination in the repeated dose (90 days) subcutaneous toxicity study of LdCen1^-/-^ in hamsters**

**Gender: Male No. of Animals/Group/Sex: 6**

| **Group &**  **Dose (mg/kg b.w.)** | **Clinical**  **Signs** | **No. of Animals Showing Clinical Signs** | | | | |
| --- | --- | --- | --- | --- | --- | --- |
|  |  | **Week** | | | | |
|  |  | **Pre-Dose** | **1** | **2** | **3** | **4** |
| G1-Placebo Control | NAD | 6 | 6 | 6 | 6 | 6 |
| G2-Vaccine 1X | NAD | 6 | 6 | 6 | 6 | 6 |
| G3-Vaccine 3X | NAD | 6 | 6 | 6 | 6 | 6 |
| G4-Placebo Control | NAD | 6 | 6 | 6 | 6 | 6 |
| G5-Vaccine 1X | NAD | 6 | 6 | 6 | 6 | 6 |
| G6-Vaccine 3X | NAD | 6 | 6 | 6 | 6 | 6 |

| **Group &**  **Dose (mg/kg b.w.)** | **Clinical**  **Signs** | **No. of Animals Showing Clinical Signs** | | | | |
| --- | --- | --- | --- | --- | --- | --- |
|  |  | **Week** | | | | |
|  |  | **5** | **6** | **7** | **8** | **9** |
| G4-Placebo Control | NAD | 6 | 6 | 6 | 6 | 6 |
| G5-Vaccine 1X | NAD | 6 | 6 | 6 | 6 | 6 |
| G6-Vaccine 3X | NAD | 6 | 6 | 6 | 6 | 6 |

| **Group &**  **Dose (mg/kg b.w.)** | **Clinical**  **Signs** | **No. of Animals Showing Clinical Signs** | | | |
| --- | --- | --- | --- | --- | --- |
|  |  | **Week** | | | |
|  |  | **10** | **11** | **12** | **13** |
| G4-Placebo Control | NAD | 6 | 6 | 6 | 6 |
| G5-Vaccine 1X | NAD | 6 | 6 | 6 | 6 |
| G6-Vaccine 3X | NAD | 6 | 6 | 6 | 6 |

**Key:** NAD-No Abnormality Detected

**Supplementary Table 4: Continued**

**Gender: Female No. of Animals/Group/Sex: 6**

| **Group &**  **Dose (mg/kg b.w.)** | **Clinical**  **Signs** | **No. of Animals Showing Clinical Signs** | | | | |
| --- | --- | --- | --- | --- | --- | --- |
|  |  | **Week** | | | | |
|  |  | **Pre-Dose** | **1** | **2** | **3** | **4** |
| G1-Placebo Control | NAD | 6 | 6 | 6 | 6 | 6 |
| G2-Vaccine 1X | NAD | 6 | 6 | 6 | 6 | 6 |
| G3-Vaccine 3X | NAD | 6 | 6 | 5 | 5 | 5 |
|  | Dullness | - | - | 1(2+) | - | - |
| G4-Placebo Control | NAD | 6 | 6 | 6 | 6 | 6 |
| G5-Vaccine 1X | NAD | 6 | 6 | 6 | 6 | 6 |
| G6-Vaccine 3X | NAD | 6 | 6 | 6 | 6 | 6 |

| **Group &**  **Dose (mg/kg b.w.)** | **Clinical**  **Signs** | **No. of Animals Showing Clinical Signs** | | | | |
| --- | --- | --- | --- | --- | --- | --- |
|  |  | **Week** | | | | |
|  |  | **5** | **6** | **7** | **8** | **9** |
| G4-Placebo Control | NAD | 5 | 5 | 5 | 5 | 5 |
|  | Alopecia | 1 | 1 | 1 | 1 | 1 |
| G5-Vaccine 1X | NAD | 6 | 6 | 6 | 6 | 6 |
| G6-Vaccine 3X | NAD | 6 | 6 | 6 | 6 | 6 |

| **Group &**  **Dose (mg/kg b.w.)** | **Clinical**  **Signs** | **No. of Animals Showing Clinical Signs** | | | |
| --- | --- | --- | --- | --- | --- |
|  |  | **Week** | | | |
|  |  | **10** | **11** | **12** | **13** |
| G4-Placebo Control | NAD | 5 | 5 | 5 | 5 |
|  | Alopecia | 1 | 1 | 1 | 1 |
| G5-Vaccine 1X | NAD | 6 | 6 | 6 | 6 |
| G6-Vaccine 3X | NAD | 6 | 6 | 6 | 6 |

**Key:** NAD-No Abnormality Detected

**Supplementary Table 5: Summary of Ophthalmic Examination in the repeated dose (90 days) subcutaneous toxicity study of LdCen1^-/-^ in hamsters**

**Gender: Male No. of Animals/Group/Sex: 6**

| **Group & Dose (mg/kg b.w.)** | **Clinical Signs** | **No. of Animals** | |
| --- | --- | --- | --- |
|  |  | **Pre-Dose** | |
|  |  | **Right Eye** | **Left Eye** |
| G1-Placebo Control | No Abnormalities Detected | 6 | 6 |
| G2-Vaccine 1X | No Abnormalities Detected | 6 | 6 |
| G3-Vaccine 3X | No Abnormalities Detected | 6 | 6 |
| G4-Placebo Control | No Abnormalities Detected | 6 | 6 |
| G5-Vaccine 1X | No Abnormalities Detected | 6 | 6 |
| G6-Vaccine 3X | No Abnormalities Detected | 6 | 6 |

| **Group & Dose (mg/kg b.w.)** | **Clinical Signs** | **No. of Animals** | |
| --- | --- | --- | --- |
|  |  | **Week 4** | |
|  |  | **Right Eye** | **Left Eye** |
| G1-Placebo Control | No Abnormalities Detected | 6 | 6 |
| G3-Vaccine 3X | No Abnormalities Detected | 6 | 6 |

| **Group & Dose (mg/kg b.w.)** | **Clinical Signs** | **No. of Animals** | |
| --- | --- | --- | --- |
|  |  | **Week 13** | |
|  |  | **Right Eye** | **Left Eye** |
| G4-Placebo Control | No Abnormalities Detected | 6 | 6 |
| G6-Vaccine 3X | No Abnormalities Detected | 6 | 6 |

**Supplementary Table 5: Continued**

**Gender: Female No. of Animals/Group/Sex: 6**

| **Group & Dose (mg/kg b.w.)** | **Clinical Signs** | **No. of Animals** | |
| --- | --- | --- | --- |
|  |  | **Pre-Dose** | |
|  |  | **Right Eye** | **Left Eye** |
| G1-Placebo Control | No Abnormalities Detected | 6 | 6 |
| G2-Vaccine 1X | No Abnormalities Detected | 6 | 6 |
| G3-Vaccine 3X | No Abnormalities Detected | 6 | 6 |
| G4-Placebo Control | No Abnormalities Detected | 6 | 6 |
| G5-Vaccine 1X | No Abnormalities Detected | 6 | 6 |
| G6-Vaccine 3X | No Abnormalities Detected | 6 | 6 |

| **Group & Dose (mg/kg b.w.)** | **Clinical Signs** | **No. of Animals** | |
| --- | --- | --- | --- |
|  |  | **Week 4** | |
|  |  | **Right Eye** | **Left Eye** |
| G1-Placebo Control | No Abnormalities Detected | 6 | 6 |
| G3-Vaccine 3X | No Abnormalities Detected | 6 | 6 |

| **Group & Dose (mg/kg b.w.)** | **Clinical Signs** | **No. of Animals** | |
| --- | --- | --- | --- |
|  |  | **Week 13** | |
|  |  | **Right Eye** | **Left Eye** |
| G4-Placebo Control | No Abnormalities Detected | 6 | 6 |
| G6-Vaccine 3X | No Abnormalities Detected | 6 | 6 |

**Supplementary Table 6: Summary of Average Food Consumption (g/day/animal) in the repeated dose (90 days) subcutaneous toxicity study of *LdCen1^-/-^* in hamsters**

**Gender: Male**

| **Day** | **Group** | | | | | | | | |
| --- | --- | --- | --- | --- | --- | --- | --- | --- | --- |
|  | **G1-Placebo Control** | | | **G2-Vaccine 1X** | | | **G3- Vaccine 3X** | | |
|  | **Mean** | **SD** | **N** | **Mean** | **SD** | **N** | **Mean** | **SD** | **N** |
| **8** | 7.04 | 0.93 | 6 | 6.91 | 0.59 | 6 | 7.00 | 1.27 | 6 |
| **15** | 6.89 | 1.02 | 6 | 6.67 | 0.88 | 6 | 6.73 | 1.26 | 6 |
| **22** | 8.04 | 1.00 | 6 | 8.90 | 2.29 | 6 | 8.04 | 1.36 | 6 |
| **30** | 6.93 | 0.88 | 6 | 7.00 | 1.00 | 6 | 7.16 | 1.89 | 6 |

| **Day** | **Group** | | | | | | | | |
| --- | --- | --- | --- | --- | --- | --- | --- | --- | --- |
|  | **G4-Placebo Control** | | | **G5-Vaccine 1X** | | | **G6- Vaccine 3X** | | |
|  | **Mean** | **SD** | **N** | **Mean** | **SD** | **N** | **Mean** | **SD** | **N** |
| **8** | 6.33 | 0.74 | 6 | 6.59 | 0.47 | 6 | 7.38 | 1.90 | 6 |
| **15** | 6.07 | 0.29 | 6 | 6.67 | 0.57 | 6 | 8.66 | 4.40 | 6 |
| **22** | 8.08 | 1.77 | 6 | 7.61 | 0.82 | 6 | 10.28 | 4.26 | 6 |
| **30** | 6.43 | 0.29 | 6 | 6.50 | 1.18 | 6 | 8.67 | 2.73 | 6 |
| **36** | 6.00 | 1.28 | 6 | 8.58↑ | 2.20 | 6 | 8.80↑ | 1.61 | 6 |
| **43** | 8.24 | 1.01 | 6 | 7.78 | 1.02 | 6 | 9.43 | 1.95 | 6 |
| **50** | 8.00 | 1.86 | 6 | 7.75 | 0.75 | 6 | 8.92 | 2.52 | 6 |
| **57** | 7.56 | 0.41 | 6 | 7.88 | 0.63 | 6 | 8.10 | 1.57 | 6 |
| **64** | 8.82 | 0.31 | 6 | 9.65 | 1.75 | 6 | 8.67 | 1.97 | 6 |
| **71** | 8.21 | 1.94 | 6 | 8.09 | 1.23 | 6 | 8.89 | 2.84 | 6 |
| **78** | 9.02 | 1.54 | 6 | 7.89 | 0.92 | 6 | 9.56 | 2.31 | 6 |
| **85** | 8.58 | 0.81 | 6 | 8.75 | 1.14 | 6 | 9.06 | 2.25 | 6 |
| **90** | 8.05 | 1.42 | 6 | 8.37 | 0.81 | 6 | 9.09 | 2.61 | 6 |

**Key:** N= No. of animals, ↑= Significantly high at P ≤0.05 as compared to G4

**Supplementary Table 6: Continued**

**Gender: Female**

| **Day** | **Group** | | | | | | | | |
| --- | --- | --- | --- | --- | --- | --- | --- | --- | --- |
|  | **G1-Placebo Control** | | | **G2-Vaccine 1X** | | | **G3- Vaccine 3X** | | |
|  | **Mean** | **SD** | **N** | **Mean** | **SD** | **N** | **Mean** | **SD** | **N** |
| **8** | 5.94 | 0.87 | 6 | 5.20 | 1.61 | 6 | 6.31 | 0.77 | 6 |
| **15** | 6.84 | 0.74 | 6 | 5.59 | 1.31 | 6 | 5.87 | 2.31 | 6 |
| **22** | 7.55 | 0.99 | 6 | 7.69 | 1.01 | 6 | 9.96 | 7.35 | 6 |
| **30** | 7.57 | 0.89 | 6 | 7.60 | 2.81 | 6 | 5.47**- | 1.01 | 5 |

| **Day** | **Group** | | | | | | | | |
| --- | --- | --- | --- | --- | --- | --- | --- | --- | --- |
|  | **G4-Placebo Control** | | | **G5-Vaccine 1X** | | | **G6- Vaccine 3X** | | |
|  | **Mean** | **SD** | **N** | **Mean** | **SD** | **N** | **Mean** | **SD** | **N** |
| **8** | 5.21 | 0.66 | 6 | 4.94 | 0.90 | 6 | 6.18 | 1.00 | 6 |
| **15** | 5.59 | 0.39 | 6 | 5.30 | 0.79 | 6 | 6.06 | 1.16 | 6 |
| **22** | 8.12 | 1.29 | 6 | 8.96 | 3.27 | 6 | 7.89 | 1.47 | 6 |
| **30** | 5.96 | 0.71 | 6 | 6.78 | 1.55 | 6 | 7.27 | 1.44 | 6 |
| **36** | 4.18 | 2.38 | 6 | 6.01 | 2.68 | 6 | 4.35 | 1.50 | 6 |
| **43** | 7.14 | 0.95 | 6 | 9.14 | 4.21 | 6 | 9.50 | 3.54 | 6 |
| **50** | 6.98 | 0.86 | 6 | 7.38 | 0.87 | 6 | 7.45 | 1.04 | 6 |
| **57** | 8.61 | 2.44 | 6 | 9.13 | 3.37 | 6 | 8.35 | 0.93 | 6 |
| **64** | 7.95 | 1.69 | 6 | 8.32 | 1.47 | 6 | 9.63 | 1.84 | 6 |
| **71** | 7.37 | 0.66 | 6 | 8.53 | 3.71 | 6 | 9.68 | 3.12 | 6 |
| **78** | 8.61 | 1.92 | 6 | 6.39 | 1.60 | 6 | 8.97 | 4.44 | 6 |
| **85** | 7.15 | 1.01 | 6 | 7.40 | 0.44 | 6 | 7.59 | 1.28 | 6 |
| **90** | 6.79 | 0.90 | 6 | 7.61 | 0.90 | 6 | 6.48 | 2.31 | 6 |

**Key:** N= No. of animals; **- = Significantly low at P ≤0.01 as compared to G1

**Supplementary Table 7: Summary of Hematology Parameters in the repeated dose (90 days) subcutaneous toxicity study of *LdCen1^-/-^* in hamsters**

**Gender: Male**

**Day: 31**

| **Parameters** | **Group & Dose (mg/kg b.w.)** | | | | | | | | |
| --- | --- | --- | --- | --- | --- | --- | --- | --- | --- |
|  | **G1-Placebo Control** | | | **G2-Vaccine 1x** | | | **G3-Vaccine 3x** | | |
|  | **Mean** | **SD** | **N** | **Mean** | **SD** | **N** | **Mean** | **SD** | **N** |
| RBC (10^6^cells/µl) | 8.43 | 0.16 | 6 | 7.76**- | 0.14 | 6 | 7.44**- | 0.41 | 6 |
| HGB (g/dl) | 17.47 | 0.36 | 6 | 15.75**- | 0.19 | 6 | 15.08**- | 0.74 | 6 |
| HCT (%) | 50.33 | 1.09 | 6 | 45.32**- | 0.69 | 6 | 43.72**- | 2.59 | 6 |
| MCV (fl) | 59.75 | 0.46 | 6 | 58.40**- | 0.56 | 6 | 58.73*- | 0.73 | 6 |
| MCH (pg) | 20.73 | 0.18 | 6 | 20.30**- | 0.19 | 6 | 20.28**- | 0.30 | 6 |
| MCHC (g/dl) | 34.68 | 0.24 | 6 | 34.77 | 0.33 | 6 | 34.55 | 0.37 | 6 |
| RDW (%) | 14.67 | 0.16 | 6 | 15.12 | 0.60 | 6 | 15.55*+ | 0.59 | 6 |
| RET | 322.28 | 59.27 | 6 | 349.93 | 85.28 | 6 | 333.02 | 71.34 | 6 |
| PLT (10^3^cells/µl) | 178.00 | 125.24 | 6 | 171.00 | 94.24 | 6 | 235.33 | 106.39 | 6 |
| MPV (fl) | 7.38 | 2.19 | 6 | 6.42 | 1.62 | 6 | 6.15 | 1.18 | 6 |
| WBC (10^3^cells/µl) | 6.72 | 1.68 | 6 | 4.52*- | 1.52 | 6 | 4.22*- | 1.06 | 6 |
| NEU (10^3^cells/µl) | 0.38 | 0.10 | 6 | 0.12**- | 0.08 | 6 | 0.17 | 0.10 | 6 |
| LYM (10^3^cells/µl) | 6.30 | 1.78 | 6 | 4.35 | 1.47 | 6 | 4.05*- | 1.00 | 6 |
| MON (10^3^cells/µl) | 0.00 | 0.00 | 6 | 0.00 | 0.00 | 6 | 0.00 | 0.00 | 6 |
| EOS (10^3^cells/µl) | 0.00 | 0.00 | 6 | 0.00 | 0.00 | 6 | 0.03 | 0.05 | 6 |
| BAS (10^3^cells/µl) | 0.00 | 0.00 | 6 | 0.00 | 0.00 | 6 | 0.00 | 0.00 | 6 |
| NEU (%) | 6.33 | 3.05 | 6 | 3.03*- | 1.51 | 6 | 3.45 | 1.37 | 6 |
| LYM (%) | 93.43 | 3.17 | 6 | 96.53 | 1.50 | 6 | 95.62 | 1.21 | 6 |
| MON (%) | 0.15 | 0.08 | 6 | 0.12 | 0.04 | 6 | 0.12 | 0.04 | 6 |
| EOS (%) | 0.08 | 0.08 | 6 | 0.32 | 0.34 | 6 | 0.82 | 0.88 | 6 |
| BAS (%) | 0.00 | 0.00 | 6 | 0.00 | 0.00 | 6 | 0.00 | 0.00 | 6 |

**Key:** N= No. of animals; *- = Significantly low at P ≤0.05 as compared to G1, **- = Significantly low at P ≤0.01 as compared to G1

**Supplementary Table 7: Continued**

**Gender: Male**

**Day: 91**

| **Parameters** | **Group & Dose (mg/kg b.w.)** | | | | | | | | |
| --- | --- | --- | --- | --- | --- | --- | --- | --- | --- |
|  | **G4-Placebo Control** | | | **G5-Vaccine 1x** | | | **G6-Vaccine 3x** | | |
|  | **Mean** | **SD** | **N** | **Mean** | **SD** | **N** | **Mean** | **SD** | **N** |
| RBC (10^6^cells/µl) | 7.84 | 0.33 | 6 | 8.32 | 0.40 | 6 | 7.64 | 0.65 | 6 |
| HGB (g/dl) | 15.83 | 0.72 | 6 | 16.77 | 1.06 | 6 | 15.25 | 1.39 | 6 |
| HCT (%) | 45.12 | 2.07 | 6 | 47.83 | 2.85 | 6 | 43.57 | 3.76 | 6 |
| MCV (fl) | 57.62 | 1.14 | 6 | 57.48 | 1.38 | 6 | 57.00 | 1.45 | 6 |
| MCH (pg) | 20.18 | 0.41 | 6 | 20.13 | 0.48 | 6 | 19.93 | 0.46 | 6 |
| MCHC (g/dl) | 35.05 | 0.40 | 6 | 35.05 | 0.30 | 6 | 34.95 | 0.28 | 6 |
| RDW (%) | 14.97 | 0.18 | 6 | 15.12 | 0.37 | 6 | 15.37 | 0.36 | 6 |
| RET | 141.00 | 37.38 | 6 | 148.08 | 22.94 | 6 | 145.70 | 24.30 | 6 |
| PLT (10^3^cells/µl) | 637.33 | 152.65 | 6 | 655.00 | 127.45 | 6 | 704.50 | 88.07 | 6 |
| MPV (fl) | 9.47 | 0.31 | 6 | 8.97 | 0.34 | 6 | 9.17 | 0.58 | 6 |
| WBC (10^3^cells/µl) | 4.46 | 2.01 | 6 | 5.25 | 1.98 | 6 | 4.65 | 1.76 | 6 |
| NEU (10^3^cells/µl) | 2.16 | 1.53 | 6 | 2.54 | 1.38 | 6 | 2.16 | 0.92 | 6 |
| LYM (10^3^cells/µl) | 2.05 | 0.71 | 6 | 2.36 | 0.76 | 6 | 2.10 | 1.04 | 6 |
| MON (10^3^cells/µl) | 0.09 | 0.04 | 6 | 0.09 | 0.04 | 6 | 0.07 | 0.02 | 6 |
| EOS (10^3^cells/µl) | 0.11 | 0.04 | 6 | 0.15 | 0.10 | 6 | 0.09 | 0.01 | 6 |
| BAS (10^3^cells/µl) | 0.01 | 0.01 | 6 | 0.02 | 0.01 | 6 | 0.02 | 0.02 | 6 |
| NEU (%) | 44.90 | 12.51 | 6 | 46.80 | 10.86 | 6 | 46.95 | 11.42 | 6 |
| LYM (%) | 49.12 | 11.29 | 6 | 46.63 | 11.29 | 6 | 45.27 | 13.06 | 6 |
| MON (%) | 2.12 | 0.98 | 6 | 1.77 | 0.67 | 6 | 1.73 | 0.78 | 6 |
| EOS (%) | 2.60 | 0.97 | 6 | 2.82 | 1.54 | 6 | 2.32 | 1.03 | 6 |
| BAS (%) | 0.27 | 0.10 | 6 | 0.40 | 0.09 | 6 | 0.35 | 0.16 | 6 |

**Key:** N= No. of animals

No statistically significant differences with placebo control found.

**Supplementary Table 7: Continued**

**Gender: Female**

**Day: 31**

| **Parameters** | **Group & Dose (mg/kg b.w.)** | | | | | | | | |
| --- | --- | --- | --- | --- | --- | --- | --- | --- | --- |
|  | **G1-Placebo Control** | | | **G2-Vaccine 1x** | | | **G3-Vaccine 3x** | | |
|  | **Mean** | **SD** | **N** | **Mean** | **SD** | **N** | **Mean** | **SD** | **N** |
| RBC (10^6^cells/µl) | 7.13 | 0.16 | 6 | 7.13 | 0.14 | 6 | 6.87*- | 0.14 | 5 |
| HGB (g/dl) | 14.83 | 0.26 | 6 | 14.85 | 0.26 | 6 | 14.40*- | 0.34 | 5 |
| HCT (%) | 43.05 | 0.94 | 6 | 42.80 | 0.76 | 5 | 41.32**- | 0.83 | 5 |
| MCV (fl) | 60.43 | 0.22 | 6 | 60.08 | 1.33 | 6 | 60.16 | 0.18 | 5 |
| MCH (pg) | 20.82 | 0.29 | 6 | 20.87 | 0.36 | 6 | 21.00 | 0.14 | 5 |
| MCHC (g/dl) | 34.47 | 0.42 | 6 | 34.75 | 0.23 | 6 | 34.88 | 0.23 | 5 |
| RDW (%) | 16.12 | 0.72 | 6 | 15.23 | 0.40 | 6 | 15.88 | 0.97 | 5 |
| RET | 321.28 | 62.16 | 6 | 308.63 | 85.27 | 6 | 229.05 | 128.63 | 6 |
| PLT (10^3^cells/µl) | 273.67 | 104.07 | 6 | 220.67 | 123.51 | 6 | 262.80 | 181.89 | 5 |
| MPV (fl) | 6.43 | 1.44 | 6 | 6.70 | 1.39 | 6 | 7.74 | 2.50 | 5 |
| WBC (10^3^cells/µl) | 6.57 | 1.05 | 6 | 5.80 | 0.72 | 6 | 4.60**- | 0.66 | 5 |
| NEU (10^3^cells/µl) | 0.23 | 0.14 | 6 | 0.25 | 0.08 | 6 | 0.10 | 0.00 | 5 |
| LYM (10^3^cells/µl) | 6.33 | 1.03 | 6 | 5.53 | 0.65 | 6 | 4.46**- | 0.64 | 5 |
| MON (10^3^cells/µl) | 0.00 | 0.00 | 6 | 0.00 | 0.00 | 6 | 0.00 | 0.00 | 5 |
| EOS (10^3^cells/µl) | 0.00 | 0.00 | 6 | 0.00 | 0.00 | 6 | 0.00 | 0.00 | 5 |
| BAS (10^3^cells/µl) | 0.00 | 0.00 | 6 | 0.00 | 0.00 | 6 | 0.00 | 0.00 | 5 |
| NEU (%) | 3.67 | 1.39 | 6 | 4.02 | 1.01 | 6 | 2.60 | 0.37 | 5 |
| LYM (%) | 96.18 | 1.43 | 6 | 95.65 | 0.91 | 6 | 96.92 | 0.13 | 5 |
| MON (%) | 0.12 | 0.08 | 6 | 0.15 | 0.05 | 6 | 0.14 | 0.05 | 5 |
| EOS (%) | 0.03 | 0.05 | 6 | 0.18 | 0.17 | 6 | 0.34 | 0.38 | 5 |
| BAS (%) | 0.00 | 0.00 | 6 | 0.00 | 0.00 | 6 | 0.00 | 0.00 | 5 |

**Key:** N= No. of animals; *- = Significantly low at P ≤0.05 as compared to G1, **- = Significantly low at P ≤0.01 as compared to G1

**Supplementary Table 7: Continued**

**Gender: Female**

**Day: 91**

| **Parameters** | **Group & Dose (mg/kg b.w.)** | | | | | | | | |
| --- | --- | --- | --- | --- | --- | --- | --- | --- | --- |
|  | **G4-Placebo Control** | | | **G5-Vaccine 1x** | | | **G6-Vaccine 3x** | | |
|  | **Mean** | **SD** | **N** | **Mean** | **SD** | **N** | **Mean** | **SD** | **N** |
| RBC (10^6^cells/µl) | 7.59 | 0.65 | 6 | 7.11 | 0.55 | 6 | 7.22 | 0.89 | 6 |
| HGB (g/dl) | 15.33 | 1.30 | 6 | 14.15 | 1.11 | 6 | 13.93 | 2.26 | 6 |
| HCT (%) | 43.67 | 3.46 | 6 | 40.65 | 3.44 | 5 | 40.42 | 6.13 | 6 |
| MCV (fl) | 57.55 | 0.79 | 6 | 57.20 | 1.21 | 6 | 55.83 | 2.45 | 6 |
| MCH (pg) | 20.22 | 0.47 | 6 | 19.88 | 0.48 | 6 | 19.22 | 0.96 | 6 |
| MCHC (g/dl) | 35.15 | 0.60 | 6 | 34.77 | 0.54 | 6 | 34.47 | 0.61 | 6 |
| RDW (%) | 15.15 | 0.27 | 6 | 15.43 | 0.27 | 6 | 15.53 | 0.52 | 6 |
| RET | 117.62 | 17.69 | 6 | 91.80 | 14.53 | 6 | 128.12 | 26.10 | 6 |
| PLT (10^3^cells/µl) | 753.83 | 123.11 | 6 | 757.17 | 132.02 | 6 | 706.17 | 159.40 | 6 |
| MPV (fl) | 9.67 | 0.29 | 6 | 9.28 | 0.23 | 6 | 9.32 | 0.68 | 6 |
| WBC (10^3^cells/µl) | 4.29 | 2.55 | 6 | 4.10 | 1.48 | 6 | 4.13 | 1.10 | 6 |
| NEU (10^3^cells/µl) | 2.18 | 2.05 | 6 | 1.87 | 0.98 | 6 | 2.08 | 0.63 | 6 |
| LYM (10^3^cells/µl) | 1.80 | 0.57 | 6 | 1.90 | 0.71 | 6 | 1.78 | 0.71 | 6 |
| MON (10^3^cells/µl) | 0.07 | 0.05 | 6 | 0.07 | 0.02 | 6 | 0.08 | 0.02 | 6 |
| EOS (10^3^cells/µl) | 0.13 | 0.07 | 6 | 0.12 | 0.06 | 6 | 0.11 | 0.05 | 6 |
| BAS (10^3^cells/µl) | 0.01 | 0.00 | 6 | 0.01 | 0.01 | 6 | 0.01 | 0.01 | 6 |
| NEU (%) | 46.05 | 14.63 | 6 | 44.47 | 8.36 | 6 | 51.32 | 9.64 | 6 |
| LYM (%) | 47.27 | 15.24 | 6 | 46.73 | 9.62 | 6 | 41.98 | 10.49 | 6 |
| MON (%) | 1.67 | 0.39 | 6 | 1.68 | 0.42 | 6 | 2.07 | 0.73 | 6 |
| EOS (%) | 3.08 | 0.75 | 6 | 2.77 | 1.11 | 6 | 2.82 | 1.61 | 6 |
| BAS (%) | 0.27 | 0.10 | 6 | 0.25 | 0.10 | 6 | 0.23 | 0.12 | 6 |

**Key:** N= No. of animals

No statistically significant differences with placebo control found.

**Supplementary Table 8: Summary of Coagulation Parameters in the repeated dose (90 days) subcutaneous toxicity study of *LdCen1^-/-^* in hamsters**

**Gender: Male**

**Day: 31**

| **Parameters** | **Group & Dose (mg/kg b.w.)** | | | | | | | | |
| --- | --- | --- | --- | --- | --- | --- | --- | --- | --- |
|  | **G1-Placebo Control** | | | **G2-Vaccine 1x** | | | **G3-Vaccine 3x** | | |
|  | **Mean** | **SD** | **N** | **Mean** | **SD** | **N** | **Mean** | **SD** | **N** |
| PT (sec) | 10.62 | 0.92 | 6 | 10.55 | 0.55 | 6 | 10.68 | 0.95 | 6 |
| APTT (sec) | 17.52 | 2.91 | 6 | 18.20 | 2.44 | 6 | 17.65 | 1.12 | 6 |
| Fibrinogen (mg/dL) | 226.62 | 58.91 | 6 | 248.00 | 56.31 | 6 | 241.08 | 143.84 | 6 |

**Gender: Male**

**Day: 91**

| **Parameters** | **Group & Dose (mg/kg b.w.)** | | | | | | | | |
| --- | --- | --- | --- | --- | --- | --- | --- | --- | --- |
|  | **G4-Placebo Control** | | | **G5-Vaccine 1x** | | | **G6-Vaccine 3x** | | |
|  | **Mean** | **SD** | **N** | **Mean** | **SD** | **N** | **Mean** | **SD** | **N** |
| PT (sec) | 10.42 | 0.90 | 6 | 10.88 | 1.02 | 6 | 10.52 | 0.63 | 6 |
| APTT (sec) | 26.00 | 2.65 | 6 | 23.28 | 3.39 | 6 | 21.08 | 5.61 | 6 |
| Fibrinogen (mg/dL) | 214.95 | 57.34 | 6 | 187.67 | 25.68 | 6 | 173.78 | 60.82 | 6 |

**Gender: Female**

**Day: 31**

| **Parameters** | **Group & Dose (mg/kg b.w.)** | | | | | | | | |
| --- | --- | --- | --- | --- | --- | --- | --- | --- | --- |
|  | **G1-Placebo Control** | | | **G2-Vaccine 1x** | | | **G3-Vaccine 3x** | | |
|  | **Mean** | **SD** | **N** | **Mean** | **SD** | **N** | **Mean** | **SD** | **N** |
| PT (sec) | 11.50 | 1.12 | 6 | 10.90 | 1.37 | 6 | 11.12 | 0.96 | 5 |
| APTT (sec) | 22.77 | 4.61 | 6 | 20.67 | 1.95 | 6 | 20.42 | 2.68 | 5 |
| Fibrinogen (mg/dL) | 200.82 | 68.05 | 6 | 204.42 | 42.31 | 6 | 180.32 | 79.61 | 5 |

**Gender: Female**

**Day: 91**

| **Parameters** | **Group & Dose (mg/kg b.w.)** | | | | | | | | |
| --- | --- | --- | --- | --- | --- | --- | --- | --- | --- |
|  | **G4-Placebo Control** | | | **G5-Vaccine 1x** | | | **G6-Vaccine 3x** | | |
|  | **Mean** | **SD** | **N** | **Mean** | **SD** | **N** | **Mean** | **SD** | **N** |
| PT (sec) | 10.17 | 1.61 | 6 | 11.38 | 1.98 | 6 | 10.93 | 0.50 | 6 |
| APTT (sec) | 23.92 | 4.87 | 6 | 22.58 | 3.33 | 6 | 27.42 | 2.91 | 6 |
| Fibrinogen (mg/dL) | 287.62 | 130.11 | 6 | 187.58 | 77.32 | 6 | 243.28 | 53.11 | 6 |

**Key:** N= No. of animals. No statistically significant differences with placebo control found.

**Supplementary Table 9: Summary of Clinical Chemistry Parameters in the repeated dose (90 days) subcutaneous toxicity study of *LdCen1^-/-^* in hamsters**

**Gender: Male**

**Day: 31**

| **Parameters** | **Group & Dose (mg/kg b.w.)** | | | | | | | | |
| --- | --- | --- | --- | --- | --- | --- | --- | --- | --- |
|  | **G1-Placebo Control** | | | **G2-Vaccine 1x** | | | **G3-Vaccine 3x** | | |
|  | **Mean** | **SD** | **N** | **Mean** | **SD** | **N** | **Mean** | **SD** | **N** |
| GLU (mg/dl) | 110.50 | 28.00 | 6 | 99.83 | 29.89 | 6 | 94.83 | 20.03 | 6 |
| TP (g/dl) | 5.82 | 0.32 | 6 | 6.10 | 0.23 | 6 | 6.18 | 0.40 | 6 |
| ALB (g/dl) | 2.77 | 0.29 | 6 | 2.83 | 0.23 | 6 | 2.92 | 0.23 | 6 |
| GLO (g/dl) | 3.03 | 0.05 | 6 | 3.25**+ | 0.08 | 6 | 3.23 | 0.34 | 6 |
| A/G Ratio | 0.90 | 0.09 | 6 | 0.88 | 0.10 | 6 | 0.93 | 0.14 | 6 |
| TRG (mg/dl) | 119.67 | 33.10 | 6 | 107.83 | 21.51 | 6 | 126.83 | 71.10 | 6 |
| ALT (U/l) | 59.33 | 14.68 | 6 | 47.83 | 13.44 | 6 | 57.17 | 6.18 | 6 |
| AST (U/l) | 69.67 | 10.80 | 6 | 64.67 | 10.11 | 6 | 73.00 | 11.24 | 6 |
| ALP (U/l) | 99.67 | 15.13 | 6 | 93.83 | 8.57 | 6 | 137.67 | 70.17 | 6 |
| BUN (mg/dl) | 17.83 | 3.13 | 6 | 19.33 | 3.56 | 6 | 19.83 | 1.47 | 6 |
| CRE (mg/dl) | 0.37 | 0.08 | 6 | 0.45 | 0.10 | 6 | 0.35 | 0.05 | 6 |

**Key:** N= No. of animals; **+ = Significantly high at P ≤0.01 as compared to G1

**Note:** The results of Bilirubin for majority of animals were below detectable range and hence Mean and SD could not be calculated.

**Supplementary Table 9: Continued**

**Gender: Male**

**Day: 91**

| **Parameters** | **Group & Dose (mg/kg b.w.)** | | | | | | | | |
| --- | --- | --- | --- | --- | --- | --- | --- | --- | --- |
|  | **G4-Placebo Control** | | | **G5-Vaccine 1x** | | | **G6-Vaccine 3x** | | |
|  | **Mean** | **SD** | **N** | **Mean** | **SD** | **N** | **Mean** | **SD** | **N** |
| GLU (mg/dl) | 85.00 | 24.10 | 6 | 101.33 | 20.58 | 6 | 104.00 | 29.47 | 6 |
| TP (g/dl) | 6.15 | 0.50 | 6 | 6.02 | 0.10 | 6 | 5.83 | 0.56 | 6 |
| ALB (g/dl) | 2.82 | 0.19 | 6 | 2.80 | 0.14 | 6 | 2.65 | 0.28 | 6 |
| GLO (g/dl) | 3.33 | 0.33 | 6 | 3.18 | 0.16 | 6 | 3.20 | 0.28 | 6 |
| A/G Ratio | 0.85 | 0.05 | 6 | 0.88 | 0.10 | 6 | 0.83 | 0.05 | 6 |
| TRG (mg/dl) | 104.00 | 36.16 | 6 | 106.67 | 27.19 | 6 | 129.50 | 30.00 | 6 |
| ALT (U/l) | 131.50 | 87.57 | 6 | 81.50 | 37.69 | 6 | 111.50 | 95.86 | 6 |
| AST (U/l) | 171.67 | 100.90 | 6 | 116.00 | 51.16 | 6 | 87.67 | 20.67 | 6 |
| ALP (U/l) | 75.00 | 14.79 | 6 | 89.33 | 15.92 | 6 | 83.00 | 11.31 | 6 |
| BUN (mg/dl) | 23.17 | 4.62 | 6 | 20.00 | 4.38 | 6 | 19.50 | 1.52 | 6 |
| CRE (mg/dl) | 0.30 | 0.06 | 6 | 0.25 | 0.08 | 6 | 0.20↓ | 0.00 | 6 |

**Key:** N= No. of animals; ↓ = Significantly low at P ≤0.05 as compared to G4

**Note:** The results of Bilirubin for majority of animals were below detectable range and hence Mean and SD could not be calculated.

**Supplementary Table 9: Continued**

**Gender: Female**

**Day: 31**

| **Parameters** | **Group & Dose (mg/kg b.w.)** | | | | | | | | |
| --- | --- | --- | --- | --- | --- | --- | --- | --- | --- |
|  | **G1-Placebo Control** | | | **G2-Vaccine 1x** | | | **G3-Vaccine 3x** | | |
|  | **Mean** | **SD** | **N** | **Mean** | **SD** | **N** | **Mean** | **SD** | **N** |
| GLU (mg/dl) | 79.67 | 12.56 | 6 | 68.17 | 10.26 | 6 | 91.20 | 5.40 | 5 |
| TP (g/dl) | 6.23 | 0.29 | 6 | 6.17 | 0.53 | 6 | 6.40 | 0.19 | 5 |
| ALB (g/dl) | 2.82 | 0.12 | 6 | 2.87 | 0.20 | 6 | 2.82 | 0.15 | 5 |
| GLO (g/dl) | 3.37 | 0.20 | 6 | 3.32 | 0.38 | 6 | 3.60 | 0.24 | 5 |
| A/G Ratio | 0.85 | 0.05 | 6 | 0.88 | 0.04 | 6 | 0.80 | 0.07 | 5 |
| TRG (mg/dl) | 153.50 | 26.53 | 6 | 130.67 | 51.47 | 6 | 113.80 | 31.39 | 5 |
| ALT (U/l) | 67.17 | 9.70 | 6 | 112.33 | 96.02 | 6 | 60.20 | 17.14 | 5 |
| AST (U/l) | 102.00 | 37.52 | 6 | 163.67 | 125.88 | 6 | 80.40 | 18.45 | 5 |
| ALP (U/l) | 127.50 | 21.40 | 6 | 114.17 | 22.39 | 6 | 149.20 | 35.71 | 5 |
| BUN (mg/dl) | 23.33 | 6.50 | 6 | 22.67 | 3.08 | 6 | 19.20 | 0.84 | 5 |
| CRE (mg/dl) | 0.42 | 0.08 | 6 | 0.33 | 0.05 | 6 | 0.32*- | 0.04 | 5 |

**Key:** N= No. of animals; *- = Significantly low at P ≤0.05 as compared to G1

**Note:** The results of Bilirubin for majority of animals were below detectable range and hence Mean and SD could not be calculated

**Supplementary Table 9: Continued**

**Gender: Female**

**Day: 91**

| **Parameters** | **Group & Dose (mg/kg b.w.)** | | | | | | | | |
| --- | --- | --- | --- | --- | --- | --- | --- | --- | --- |
|  | **G4-Placebo Control** | | | **G5-Vaccine 1x** | | | **G6-Vaccine 3x** | | |
|  | **Mean** | **SD** | **N** | **Mean** | **SD** | **N** | **Mean** | **SD** | **N** |
| GLU (mg/dl) | 71.17 | 14.33 | 6 | 91.00 | 15.56 | 6 | 83.83 | 10.59 | 6 |
| TP (g/dl) | 6.07 | 0.38 | 6 | 6.18 | 0.41 | 6 | 5.98 | 0.10 | 6 |
| ALB (g/dl) | 2.72 | 0.13 | 6 | 2.67 | 0.20 | 6 | 2.65 | 0.08 | 6 |
| GLO (g/dl) | 3.35 | 0.35 | 6 | 3.53 | 0.33 | 6 | 3.33 | 0.10 | 6 |
| A/G Ratio | 0.83 | 0.10 | 6 | 0.75 | 0.10 | 6 | 0.78 | 0.04 | 6 |
| TRG (mg/dl) | 122.00 | 75.37 | 6 | 129.00 | 43.23 | 6 | 107.33 | 23.80 | 6 |
| ALT (U/l) | 54.33 | 15.36 | 6 | 74.67 | 30.28 | 6 | 68.00 | 16.15 | 6 |
| AST (U/l) | 75.00 | 9.98 | 6 | 98.67 | 35.90 | 6 | 94.17 | 14.05 | 6 |
| ALP (U/l) | 105.67 | 17.28 | 6 | 113.33 | 13.13 | 6 | 109.00 | 16.83 | 6 |
| BUN (mg/dl) | 30.33 | 5.43 | 6 | 27.50 | 8.50 | 6 | 25.50 | 2.74 | 6 |
| CRE (mg/dl) | 0.28 | 0.08 | 6 | 0.23 | 0.05 | 6 | 0.22 | 0.04 | 5 |

**Key:** N= No. of animals

**Note:** The results of Bilirubin for majority of animals were below detectable range and hence Mean and SD could not be calculated

No statistically significant differences with placebo control found.

**Supplementary Table** **10: Summary of Urinalysis Parameters in the repeated dose (90 days) subcutaneous toxicity study of *LdCen1^-/-^* in hamsters**

**Gender: Male No. of Animals/Group/Sex: 6**

**Day: 31**

| **Parameters** | **Group & Dose (mg/kg b.w.)** | | |
| --- | --- | --- | --- |
|  | **G1-Placebo** | **G2-Vaccine 1x** | **G3-Vaccine 3x** |
| **Colour** | | | |
| Light Yellow | - | 2 | - |
| Yellow | 4 | 3 | 1 |
| Dark Yellow | 2 | 1 | 5 |
| **Appearance/Clarity** | | | |
| Cloudy | 6 | 6 | 6 |
| **Specific Gravity** | | | |
| 1.010 | 2 | - | - |
| 1.015 | 4 | 6 | 6 |
| **Nitrite** | | | |
| Negative | 6 | 6 | 6 |
| **pH** | | | |
| Mean ± SD | 8.33 ± 0.98 | 8.00 ± 0.84 | 8.00 ± 1.10 |
| **Protein (mg/dl)** | | | |
| 100 | 6 | 1 | 3 |
| 300 | - | - | 3 |
| 1000 | - | 5 | - |
| **Glucose (mg/dl)** | | | |
| Negative | 6 | 6 | 6 |
| **Ketone bodies (mg/dl)** | | | |
| Negative | 2 | 3 | - |
| 5 | 1 | 1 | 3 |
| 10 | 3 | 2 | 3 |
| **Urobilinogen (EU/dl)** | | | |
| Normal | 6 | 6 | 6 |
| **Bilirubin (mg/dl)** | | | |
| Negative | 3 | 3 | 2 |
| 0.5 | 3 | 3 | 4 |

All parameters are within the normal range

**Supplementary Table 10: Continued**

**Gender: Male No. of Animals/Group/Sex: 6**

**Day: 31**

| **Parameters** | | **Group & Dose (mg/kg b.w.)** | | | | |
| --- | --- | --- | --- | --- | --- | --- |
|  |  | **G1-Placebo** | | **G2-Vaccine 1x** | | **G3-Vaccine 3x** |
| **Blood (RBC/µl)** | | | | | | |
| Negative | 6 | | 6 | | 6 | |
| **Leucocytes (WBC/µl)** | | | | | | |
| Negative | 3 | | 2 | | 1 | |
| 10 | 3 | | 4 | | 5 | |
| **Microscopy** | | | | | | |
| **Epithelial Cells** | | | | | | |
| 0 (0/hpf) | | 4 | | 2 | | 1 |
| 1+ (1-2/hpf) | | 2 | | 4 | | 5 |
| **Crystals** | | | | | | |
| 1+ (1-5/hpf) | | 6 | | 6 | | 5 |
| **Other Abnormal Constituents** | | | | | | |
| Nil | | 6 | | 6 | | 6 |

All parameters are within the normal range

**Supplementary Table 10: Continued**

**Gender: Male No. of Animals/Group/Sex: 6**

**Day: 91**

| **Parameters** | **Group & Dose (mg/kg b.w.)** | | |
| --- | --- | --- | --- |
|  | **G4-Placebo** | **G5-Vaccine 1x** | **G6-Vaccine 3x** |
| **Colour** | | | |
| Light Yellow | 4 | 6 | - |
| Yellow | 2 | - | 6 |
| **Appearance/Clarity** | | | |
| Clear | 4 | 5 | - |
| Slightly cloudy | 2 | 1 | 6 |
| **Specific Gravity** | | | |
| <=1.005 | 1 | - | - |
| 1.010 | 3 | 6 |  |
| 1.015 | 2 | - | 6 |
| **Nitrite** | | | |
| Negative | 6 | 6 | 6 |
| **pH** |  | | |
| Mean ± SD | 8.17 ± 0.68 | 8.42 ± 0.20 | 9.00 ± 0.00↑↑ |
| **Protein (mg/dl)** | | | |
| Negative | 4 | 5 | - |
| 30 | 1 | 1 | 1 |
| 100 | 1 | - | 5 |
| **Glucose (mg/dl)** | | | |
| Negative | 6 | 6 | 6 |
| **Ketone bodies (mg/dl)** | | | |
| Negative | 4 | 6 | - |
| 5 | - | - | - |
| 10 | 2 | - | 5 |
| 50 | - | - | 1 |
| **Urobilinogen (EU/dl)** | | | |
| Normal | 6 | 6 | 6 |
| **Bilirubin (mg/dl)** |  | | |
| Negative | 5 | 6 | 5 |
| 0.5 | 1 | - | 1 |

Key: ↑↑= Significantly high at P ≤0.01 as compared to G4

**Supplementary Table 10: Continued**

**Gender: Male No. of Animals/Group/Sex: 6**

**Day: 91**

| **Parameters** | **Group & Dose (mg/kg b.w.)** | | | | |
| --- | --- | --- | --- | --- | --- |
|  | **G4-Placebo** | **G5-Vaccine 1x** | | **G6-Vaccine 3x** | |
| **Blood (RBC/µl)** | | | | | |
| Negative | 3 | | 6 | | 6 |
| 10 | 2 | | - | | - |
| 50 | 1 | | - | | - |
| **Leucocytes (WBC/µl)** | | | | | |
| Negative | 6 | | 6 | | 6 |
| 10 | - | | - | | - |
| **Microscopy** | | | | | |
| **Epithelial Cells** | | | | | |
| 0 (0/hpf) | - | - | | - | |
| 1+ (1-2/hpf) | 5 | 6 | | 5 | |
| 2+ (3-4/hpf) | 1 | - | | 1 | |
| **Crystals** | | | | | |
| 0 (0/hpf) | - | - | | - | |
| 1+ (1-5/hpf) | 4 | 6 | | 5 | |
| 2+ (5-10/hpf) | 2 | - | | 1 | |
| **Other Abnormal Constituents** | | | | | |
| Nil | 6 | 6 | | 6 | |

All parameters are within the normal range

**Supplementary Table 10: Continued**

**Gender: Female No. of Animals/Group/Sex: 6**

**Day: 31**

| **Parameters** | **Group & Dose (mg/kg b.w.)** | | |
| --- | --- | --- | --- |
|  | **G1-Placebo** | **G2-Vaccine 1x** | **G3-Vaccine 3x** |
| **Colour** | | | |
| Yellow | 2 | 2 | 2 |
| Dark Yellow | 4 | 2 | 3 |
| Dark Orange | - | 2 | - |
| **Appearance/Clarity** | | | |
| Clear | 6 | - | - |
| Cloudy | - | 6 | 5 |
| **Specific Gravity** | | | |
| 1.010 | 1 | 1 | 2 |
| 1.015 | 3 | 3 | 3 |
| 1.020 | 2 | - | - |
| 1.025 | - | 2 | - |
| **Nitrite** | | | |
| Negative | 6 | 6 | 5 |
| **pH** | | | |
| Mean ± SD | 7.67 ± 1.29 | 7.26 ± 1.44 | 8.10 ± 0.96 |
| **Protein (mg/dl)** | | | |
| Negative | 1 | - | - |
| 10 | - | - | 1 |
| 30 | - | - | - |
| 100 | - | 3 | 3 |
| 300 | 2 | - | 1 |
| 1000 | 3 | 3 | - |
| **Glucose (mg/dl)** | | | |
| Negative | 6 | 6 | 5 |
| **Ketone bodies (mg/dl)** | | | |
| Negative | - | 2 | 2 |
| 5 | 5 | 2 | 3 |
| 10 | 1 | 1 | - |
| 50 | - | 1 | - |
| **Urobilinogen (EU/dl)** | | | |
| Normal | 6 | 6 | 5 |

**Supplementary Table 10: Continued**

**Gender: Female No. of Animals/Group/Sex: 6**

**Day: 31**

| **Parameters** | | **Group & Dose (mg/kg b.w.)** | | | | |
| --- | --- | --- | --- | --- | --- | --- |
|  |  | **G1-Placebo** | | **G2-Vaccine 1x** | | **G3-Vaccine 3x** |
| **Bilirubin (mg/dl)** | | | | | | |
| 0.5 | 4 | | 4 | | 3 | |
| 1 | 2 | | 2 | | 2 | |
| **Blood (RBC/µl)** | | | | | | |
| Negative | 6 | | 6 | | 5 | |
| **Leucocytes (WBC/µl)** | | | | | | |
| Negative | 1 | | 3 | | 3 | |
| 10 | 4 | | 3 | | 1 | |
| 25 | 1 | | - | | 1 | |
| **Microscopy** | | | | | | |
| **Epithelial Cells** | | | | | | |
| 0 (0/hpf) | | 1 | | 3 | | - |
| 1+ (1-2/hpf) | | 5 | | 3 | | 5 |
| **Crystals** | | | | | | |
| 1+ (1-5/hpf) | | 5 | | 5 | | 3 |
| 2+ (5-10/hpf) | | 1 | | 1 | | 2 |
| **Other Abnormal Constituents** | | | | | | |
| Nil | | 6 | | 6 | | 5 |

All parameters are within the normal range

**Supplementary Table 10: Continued**

**Gender: Female No. of Animals/Group/Sex: 6**

**Day: 91**

| **Parameters** | **Group & Dose (mg/kg b.w.)** | | |
| --- | --- | --- | --- |
|  | **G4-Placebo** | **G5-Vaccine 1x** | **G6-Vaccine 3x** |
| **Colour** |  | | |
| Light Yellow | 6 | 5 | 6 |
| Yellow | - | 1 | - |
| **Appearance/Clarity** |  | | |
| Clear | 6 | 4 | - |
| Slightly cloudy | - | 2 | 6 |
| **Specific Gravity** |  | | |
| <=1.005 | 2 | - | 2 |
| 1.010 | 3 | 5 | 4 |
| 1.015 | 1 | 1 | - |
| **Nitrite** |  | | |
| Negative | 6 | 6 | 6 |
| **pH** |  | | |
| Mean ± SD | 7.67 ± 0.52 | 8.17 ± 0.41 | 7.50 ± 0.00 |
| **Protein (mg/dl)** |  | | |
| Negative | 5 | 4 | - |
| 10 | 1 | - | - |
| 30 | - | 1 | - |
| 100 | - | 1 | 6 |
| **Glucose (mg/dl)** |  | | |
| Negative | 6 | 6 | 6 |
| **Ketone bodies (mg/dl)** |  | | |
| Negative | 6 | 6 | 6 |
| **Urobilinogen (EU/dl)** | | | |
| Normal | 6 | 6 | 6 |

**Supplementary Table 10: Continued**

**Gender: Female No. of Animals/Group/Sex: 6**

**Day: 91**

| **Parameters** | **Group & Dose (mg/kg b.w.)** | | | | |
| --- | --- | --- | --- | --- | --- |
|  | **G4-Placebo** | **G5-Vaccine 1x** | | **G6-Vaccine 3x** | |
| **Bilirubin (mg/dl)** | | | | | |
| 0.5 | 6 | | 6 | | 6 |
| 1 | - | | - | | - |
| **Blood (RBC/µl)** | | | | | |
| Negative | 3 | | 6 | | 6 |
| 5 | 1 | | - | | - |
| 50 | 2 | | - | | - |
| **Leucocytes (WBC/µl)** | | | | | |
| Negative | 6 | | 6 | | 6 |
| 10 | - | | - | | - |
| 25 | - | | - | | - |
| **Microscopy** | | | | | |
| **Epithelial Cells** |  | | | | |
| 0 (0/hpf) | - | - | | - | |
| 1+ (1-2/hpf) | 2 | 4 | | 3 | |
| 2+ (3-4/hpf) | 4 | 2 | | 3 | |
| **Crystals** | | | | | |
| 0 (0/hpf) | - | - | | - | |
| 1+ (1-5/hpf) | 6 | 5 | | 3 | |
| 2+ (5-10/hpf) | - | 1 | | 3 | |
| **Other Abnormal Constituents** | | | | | |
| Nil | 6 | 6 | | 6 | |

All parameters are within the normal range

**Supplementary Table 11: Summary of Absolute Organ Weight (g) in the repeated dose (90 days) subcutaneous toxicity study of *LdCen1^-/-^* in hamsters**

**Gender: Male**

**Day: 31**

| **Parameters** | **Group & Dose (mg/kg b.w.)** | | | | | | | | |
| --- | --- | --- | --- | --- | --- | --- | --- | --- | --- |
|  | **G1-Placebo Control** | | | **G2-Vaccine 1x** | | | **G3-Vaccine 3x** | | |
|  | **Mean** | **SD** | **N** | **Mean** | **SD** | **N** | **Mean** | **SD** | **N** |
| Terminal Body Weight | 129.15 | 15.51 | 6 | 127.78 | 12.87 | 6 | 131.10 | 17.96 | 6 |
| Liver | 4.0475 | 0.5894 | 6 | 4.2089 | 0.6643 | 6 | 4.2937 | 0.4598 | 6 |
| Kidneys | 0.9738 | 0.1172 | 6 | 0.9905 | 0.2245 | 6 | 0.9857 | 0.1464 | 6 |
| Heart | 0.5513 | 0.0560 | 6 | 0.5746 | 0.0630 | 6 | 0.6087 | 0.0349 | 6 |
| Brain | 1.0214 | 0.0706 | 6 | 1.0444 | 0.0381 | 6 | 1.0418 | 0.0483 | 6 |
| Thymus | 0.0440 | 0.0189 | 6 | 0.0374 | 0.0148 | 6 | 0.0518 | 0.0296 | 6 |
| Adrenals | 0.0359 | 0.0046 | 6 | 0.0376 | 0.0101 | 6 | 0.0363 | 0.0018 | 6 |
| Epididymides | 1.3475 | 0.0429 | 6 | 1.3627 | 0.1662 | 6 | 1.3227 | 0.1570 | 6 |
| Testes | 2.7182 | 0.2414 | 6 | 2.9441 | 0.5700 | 6 | 2.9878 | 0.3958 | 6 |

**Day: 91**

| **Parameters** | **Group & Dose (mg/kg b.w.)** | | | | | | | | |
| --- | --- | --- | --- | --- | --- | --- | --- | --- | --- |
|  | **G4-Placebo Control** | | | **G5-Vaccine 1x** | | | **G6-Vaccine 3x** | | |
|  | **Mean** | **SD** | **N** | **Mean** | **SD** | **N** | **Mean** | **SD** | **N** |
| Terminal Body Weight | 131.42 | 10.05 | 6 | 129.86 | 6.60 | 6 | 132.26 | 15.58 | 6 |
| Liver | 3.8784 | 0.2037 | 6 | 4.1046 | 0.5697 | 6 | 4.5299 | 0.6865 | 6 |
| Kidneys | 1.0280 | 0.0809 | 6 | 1.1139 | 0.1110 | 6 | 1.1538 | 0.0877 | 6 |
| Heart | 0.5930 | 0.0426 | 6 | 0.6539 | 0.0873 | 6 | 0.6132 | 0.0442 | 6 |
| Brain | 1.1429 | 1.1389 | 6 | 1.2160 | 0.0656 | 6 | 1.1447 | 0.0457 | 6 |
| Thymus | 0.0461 | 0.0191 | 6 | 0.0392 | 0.0092 | 6 | 0.0351 | 0.0102 | 6 |
| Adrenals | 0.0302 | 0.0073 | 6 | 0.0329 | 0.0050 | 6 | 0.0328 | 0.0031 | 6 |
| Epididymides | 1.1671 | 0.1195 | 6 | 1.2374 | 0.1710 | 6 | 1.2209 | 0.2552 | 6 |
| Testes | 2.9495 | 0.6277 | 6 | 3.0166 | 0.4390 | 6 | 2.7949 | 0.9237 | 6 |

**Key:** N=No. of animals; No statistically significant differences anywhere.

**Supplementary Table 11: Continued**

**Gender: Female**

**Day: 31**

| **Parameters** | **Group & Dose (mg/kg b.w.)** | | | | | | | | |
| --- | --- | --- | --- | --- | --- | --- | --- | --- | --- |
|  | **G1-Placebo Control** | | | **G2-Vaccine 1x** | | | **G3-Vaccine 3x** | | |
|  | **Mean** | **SD** | **N** | **Mean** | **SD** | **N** | **Mean** | **SD** | **N** |
| Terminal Body Weight | 124.32 | 9.69 | 6 | 121.45 | 19.13 | 6 | 123.94 | 18.39 | 5 |
| Liver | 4.1880 | 0.4309 | 6 | 4.2695 | 0.9602 | 6 | 3.8663 | 0.6464 | 5 |
| Kidneys | 1.1584 | 0.2119 | 6 | 1.0882 | 0.1093 | 6 | 1.1327 | 0.2393 | 5 |
| Heart | 0.6385 | 0.1080 | 6 | 0.6151 | 0.0982 | 6 | 0.5747 | 0.0939 | 5 |
| Brain | 1.0788 | 0.0701 | 6 | 1.0548 | 0.0503 | 6 | 1.0746 | 0.0599 | 5 |
| Thymus | 0.0399 | 0.0196 | 6 | 0.0441 | 0.0274 | 6 | 0.0582 | 0.0242 | 5 |
| Adrenals | 0.0212 | 0.0044 | 6 | 0.0203 | 0.0070 | 6 | 0.0209 | 0.0071 | 5 |
| Ovaries | 0.0825 | 0.0120 | 6 | 0.0829 | 0.0256 | 6 | 0.0917 | 0.0224 | 5 |
| Uterus with Cervix | 0.6277 | 0.1646 | 6 | 0.5459 | 0.1327 | 6 | 0.5626 | 0.0929 | 5 |

**Day: 91**

| **Parameters** | **Group & Dose (mg/kg b.w.)** | | | | | | | | |
| --- | --- | --- | --- | --- | --- | --- | --- | --- | --- |
|  | **G4-Placebo Control** | | | **G5-Vaccine 1x** | | | **G6-Vaccine 3x** | | |
|  | **Mean** | **SD** | **N** | **Mean** | **SD** | **N** | **Mean** | **SD** | **N** |
| Terminal Body Weight | 119.80 | 13.08 | 6 | 108.84 | 3.96 | 6 | 119.29 | 9.92 | 6 |
| Liver | 4.1690 | 0.4392 | 6 | 3.9381 | 0.3430 | 6 | 4.0773 | 0.4533 | 6 |
| Kidneys | 1.2029 | 0.1250 | 6 | 1.2356 | 0.2790 | 6 | 1.2966 | 0.1764 | 6 |
| Heart | 0.7338 | 0.1796 | 6 | 0.5639 | 0.0644 | 6 | 0.6538 | 0.1959 | 6 |
| Brain | 1.0743 | 0.1022 | 6 | 1.1115 | 0.0493 | 6 | 1.1166 | 0.0308 | 6 |
| Thymus | 0.0581 | 0.0284 | 6 | 0.0406 | 0.0068 | 6 | 0.0375 | 0.0198 | 6 |
| Adrenals | 0.0208 | 0.0052 | 6 | 0.0208 | 0.0049 | 6 | 0.0223 | 0.0046 | 6 |
| Ovaries | 0.0408 | 0.0102 | 6 | 0.0393 | 0.0088 | 6 | 0.0454 | 0.0131 | 6 |
| Uterus with Cervix | 0.7811 | 0.3662 | 6 | 0.7357 | 0.2647 | 6 | 0.8564 | 0.2045 | 6 |

**Key:** N=No. of animals; No statistically significant differences anywhere.

**Supplementary Table 12: Summary of Relative Organ Weight (%) in the repeated dose (90 days) subcutaneous toxicity study of *LdCen1^-/-^* in hamsters**

**Gender: Male**

**Day: 31**

| **Parameters** | **Group & Dose (mg/kg b.w.)** | | | | | | | | |
| --- | --- | --- | --- | --- | --- | --- | --- | --- | --- |
|  | **G1-Placebo Control** | | | **G2-Vaccine 1x** | | | **G3-Vaccine 3x** | | |
|  | **Mean** | **SD** | **N** | **Mean** | **SD** | **N** | **Mean** | **SD** | **N** |
| Liver | 3.1348 | 0.2661 | 6 | 3.2979 | 0.4611 | 6 | 3.3072 | 0.4156 | 6 |
| Kidneys | 0.7610 | 0.1163 | 6 | 0.7771 | 0.1745 | 6 | 0.7559 | 0.1016 | 6 |
| Heart | 0.4286 | 0.0360 | 6 | 0.4506 | 0.0359 | 6 | 0.4720 | 0.0744 | 6 |
| Brain | 0.7966 | 0.0713 | 6 | 0.8232 | 0.0741 | 6 | 0.8049 | 0.0965 | 6 |
| Thymus | 0.0335 | 0.0121 | 6 | 0.0300 | 0.0143 | 6 | 0.0396 | 0.0216 | 6 |
| Adrenals | 0.0279 | 0.0038 | 6 | 0.0297 | 0.0086 | 6 | 0.0281 | 0.0042 | 6 |
| Epididymides | 1.0549 | 0.1232 | 6 | 1.0651 | 0.0558 | 6 | 1.0123 | 0.0598 | 6 |
| Testes | 2.1340 | 0.3497 | 6 | 2.3159 | 0.4586 | 6 | 2.3014 | 0.3309 | 6 |

**Day: 91**

| **Parameters** | **Group & Dose (mg/kg b.w.)** | | | | | | | | |
| --- | --- | --- | --- | --- | --- | --- | --- | --- | --- |
|  | **G4-Placebo Control** | | | **G5-Vaccine 1x** | | | **G6-Vaccine 3x** | | |
|  | **Mean** | **SD** | **N** | **Mean** | **SD** | **N** | **Mean** | **SD** | **N** |
| Liver | 2.9574 | 0.1397 | 6 | 3.1570 | 0.3718 | 6 | 3.4490 | 0.5819 | 6 |
| Kidneys | 0.7847 | 0.0717 | 6 | 0.8575 | 0.0710 | 6 | 0.8786 | 0.0904 | 6 |
| Heart | 0.4540 | 0.0540 | 6 | 0.5024 | 0.0481 | 6 | 0.4689 | 0.0645 | 6 |
| Brain | 0.8626 | 0.1165 | 6 | 0.8414 | 0.0507 | 6 | 0.8738 | 0.0938 | 6 |
| Thymus | 0.0354 | 0.0155 | 6 | 0.0303 | 0.0074 | 6 | 0.0263 | 0.0049 | 6 |
| Adrenals | 0.0233 | 0.0064 | 6 | 0.0254 | 0.0039 | 6 | 0.0249 | 0.0020 | 6 |
| Epididymides | 0.8942 | 0.1284 | 6 | 0.9530 | 0.1272 | 6 | 0.9196 | 0.1351 | 6 |
| Testes | 2.2752 | 0.5850 | 6 | 2.3348 | 0.4132 | 6 | 2.1010 | 0.6313 | 6 |

**Key:** N=No. of animals; No statistically significant differences anywhere.

**Supplementary Table: 12: Continued**

**Gender: Female**

**Day: 31**

| **Parameters** | **Group & Dose (mg/kg b.w.)** | | | | | | | | |
| --- | --- | --- | --- | --- | --- | --- | --- | --- | --- |
|  | **G1-Placebo Control** | | | **G2-Vaccine 1x** | | | **G3-Vaccine 3x** | | |
|  | **Mean** | **SD** | **N** | **Mean** | **SD** | **N** | **Mean** | **SD** | **N** |
| Liver | 3.3744 | 0.3113 | 6 | 3.6047 | 1.1258 | 6 | 3.1161 | 0.2088 | 5 |
| Kidneys | 0.9290 | 0.1314 | 6 | 0.9175 | 0.2011 | 6 | 0.9142 | 0.1273 | 5 |
| Heart | 0.5139 | 0.0784 | 6 | 0.5077 | 0.0395 | 6 | 0.4606 | 0.0910 | 5 |
| Brain | 0.8691 | 0.0397 | 6 | 0.8873 | 0.1547 | 6 | 0.8792 | 0.1062 | 5 |
| Thymus | 0.0327 | 0.0172 | 6 | 0.0355 | 0.0311 | 6 | 0.0472 | 0.0177 | 5 |
| Adrenals | 0.0170 | 0.0027 | 6 | 0.0168 | 0.0050 | 6 | 0.0171 | 0.0056 | 5 |
| Ovaries | 0.0662 | 0.0070 | 6 | 0.0678 | 0.0147 | 6 | 0.0745 | 0.0157 | 5 |
| Uterus with Cervix | 0.5034 | 0.1200 | 6 | 0.4475 | 0.0675 | 6 | 0.4606 | 0.0910 | 5 |

**Day: 91**

| **Parameters** | **Group & Dose (mg/kg b.w.)** | | | | | | | | |
| --- | --- | --- | --- | --- | --- | --- | --- | --- | --- |
|  | **G4-Placebo Control** | | | **G5-Vaccine 1x** | | | **G6-Vaccine 3x** | | |
|  | **Mean** | **SD** | **N** | **Mean** | **SD** | **N** | **Mean** | **SD** | **N** |
| Liver | 3.5299 | 0.6243 | 6 | 3.6243 | 0.3554 | 6 | 3.4203 | 0.2977 | 6 |
| Kidneys | 1.0128 | 0.1354 | 6 | 1.1348 | 0.2539 | 6 | 1.0841 | 0.0848 | 6 |
| Heart | 0.6244 | 0.1853 | 6 | 0.5175 | 0.0495 | 6 | 0.5463 | 0.1424 | 6 |
| Brain | 0.9121 | 0.1863 | 6 | 1.0214 | 0.0335 | 6 | 0.9414 | 0.0820 | 6 |
| Thymus | 0.0501 | 0.0279 | 6 | 0.0374 | 0.0073 | 6 | 0.0316 | 0.0168 | 6 |
| Adrenals | 0.0180 | 0.0071 | 6 | 0.0191 | 0.0044 | 6 | 0.0187 | 0.0032 | 6 |
| Ovaries | 0.0348 | 0.0106 | 6 | 0.0364 | 0.0091 | 6 | 0.0383 | 0.0116 | 6 |
| Uterus with Cervix | 0.6544 | 0.2915 | 6 | 0.6760 | 0.2441 | 6 | 0.7147 | 0.1471 | 6 |

**Key:** N=No. of animals; No statistically significant differences anywhere.

**Supplementary Table 13: Summary of Gross Pathology Findings in the repeated dose (90 days) subcutaneous toxicity study of *LdCen1^-/-^* in hamsters**

**Gender: Male**

| **Gross Pathology observation (s)** | **Number of animals with or without lesion (s)/ Numbers of animals observed** | | | | | |
| --- | --- | --- | --- | --- | --- | --- |
|  | **G1** | **G2** | **G3** | **G4** | **G5** | **G6** |
| No abnormalities detected | 6/6 | 6/6 | 5/6 | 6/6 | 6/6 | 6/6 |
| Testes and epididymides - Adhesion, unilateral | 0/6 | 0/6 | 1/6 | 0/6 | 0/6 | 0/6 |
| Seminal vesicle - Small, unilateral | 0/6 | 0/6 | 1/6 | 0/6 | 0/6 | 0/6 |

**Gender: Female**

| **Gross Pathology observation (s)** | **Number of animals with or without lesion (s)/ Numbers of animals observed** | | | | | |
| --- | --- | --- | --- | --- | --- | --- |
|  | **G1** | **G2** | **G3** | **G4** | **G5** | **G6** |
| No abnormalities detected | 6/6 | 6/6 | 4/6 | 6/6 | 6/6 | 6/6 |
| Ovary – Enlarged, dark red in colour, unilateral | 0/6 | 0/6 | 1/6 | 0/6 | 0/6 | 0/6 |
| Thymus – Reduced; Eyes – Right side, black, pus was oozed out from cut open lachrymal gland; Lower jaw – Right side, near salivary gland, blackish enlargement; Brain – cerebrum, right side, abscess | 0/6 | 0/6 | 1/6* | 0/6 | 0/6 | 0/6 |

*: Found dead animal

**Supplementary Table 14: Summary of Histopathology Findings in the repeated dose (90 days) subcutaneous toxicity study of *LdCen1^-/-^* in hamsters**

**Gender: Male No. of Animals/Group/Sex: 6**

| **Organs/**  **Histopathology** | **No. of tissues showing lesion / no. of tissues examined** | |
| --- | --- | --- |
|  | **G4** | **G6** |
| **LIVER (N)** | 4/6 | 3/6 |
| Infiltration, MNC, multifocal | 2/6 | 2/6 |
| Cyst, bile duct, focal | 0/6 | 1/6 |
| **LUNGS with bronchi and bronchiole (N)** | 6/6 | 6/6 |
| **KIDNEYS (N)** | 4/6 | 5/6 |
| Dilatation, tubule, focal | 1/6 | 0/6 |
| Basophilia, tubule, multifocal | 1/6 | 1/6 |
| **URINARY BLADDER (N)** | 6/6 | 6/6 |
| **SPLEEN (N)** | 6/6 | 6/6 |
| **THYMUS (N)** | 3/6 | 4/6 |
| Age-related involution | 1/6 | 0/6 |
| Absent* | 2/6 | 2/6 |
| **MESENTERIC LYMPHNODES (N)** | 6/6 | 6/6 |
| **PITUITARY GLAND (N)** | 4/6 | 4/6 |
| Vacuolation, pars distalis | 2/6 | 1/6 |
| Cyst, pars distalis | 0/6 | 1/6 |
| Pseudo cyst, pars distalis | 0/6 | 1/6 |
| **TESTES (N)** | 6/6 | 6/6 |
| **ADRENAL GLANDS (N)** | 6/6 | 6/6 |
| **EPIDIDYMIDES (N)** | 6/6 | 6/6 |
| **PROSTATE (N)** | 6/6 | 6/6 |
| **SEMINAL VESICLES with coagulating glands (N)** | 6/6 | 6/6 |
| **HEART (N)** | 6/6 | 6/6 |
| **AORTA (N)** | 6/6 | 6/6 |
| **SALIVARY GLANDS (N)** | 6/6 | 6/6 |
| **MANDIBULAR LYMPHNODES (N)** | 6/6 | 6/6 |
| **ESOPHAGUS (N)** | 6/6 | 6/6 |
| **TRACHEA (N)** | 6/6 | 6/6 |
| **THYROID (N)** | 4/6 | 4/6 |
| Cystic follicle, focal | 2/6 | 2/6 |
| **PARATHYROID (N)** | 6/6 | 6/6 |

**Key:** N –within normal histological limits; MNC – Mononuclear Cell; PMNC – Polymorpho-nuclear cell; * - Thymus was absent or replaced with fat might be due to age related complete involution.

**Supplementary Table 14: Continued**

**Gender: Male No. of Animals/Group/Sex: 6**

| **Organs/**  **Histopathology** | **No. of tissues showing lesion / no. of tissues examined** | |
| --- | --- | --- |
|  | **G4** | **G6** |
| **STOMACH (N)** | 6/6 | 6/6 |
| **PANCREAS (N)** | 6/6 | 6/6 |
| **DUODENUM (N)** | 6/6 | 6/6 |
| **JEJUNUM (N)** | 6/6 | 6/6 |
| **ILEUM with Peyer’s patches (N)** | 6/6 | 6/6 |
| **CECUM (N)** | 6/6 | 6/6 |
| **COLON (N)** | 6/6 | 6/6 |
| **RECTUM (N)** | 6/6 | 6/6 |
| **BRAIN (N)** | 6/6 | 6/6 |
| **SCIATIC NERVE (N)** | 6/6 | 6/6 |
| **EYES with optic nerve (N)** | 6/6 | 6/6 |
| **SKIN (N)** | 6/6 | 6/6 |
| **MAMMARY GLANDS (N)** | 6/6 | 6/6 |
| **SKELETAL MUSCLE (N)** | 6/6 | 6/6 |
| **SPINAL CORD (N)** | 6/6 | 6/6 |
| **FEMUR WITH JOINT (N)** | 6/6 | 6/6 |
| **SITE OF INJECTION (N)** | 6/6 | 6/6 |

**Key:** N –within normal histological limits; MNC – Mononuclear Cell; PMNC – Polymorpho-nuclear cell

**Supplementary Table 14: Continued**

**Gender: Female No. of Animals/Group/Sex: 6**

| **Organs/**  **Histopathology** | **No. of tissues showing lesion / no. of tissues examined** | |
| --- | --- | --- |
|  | **G4** | **G6** |
| **LIVER (N)** | 2/6 | 3/6 |
| Infiltration, MNC, multifocal | 4/6 | 1/6 |
| Cyst, bile duct, focal | 0/6 | 2/6 |
| **LUNGS with bronchi and bronchiole (N)** | 6/6 | 6/6 |
| **KIDNEYS (N)** | 3/6 | 4/6 |
| Basophilia, tubule, multifocal | 1/6 | 0/6 |
| CPN, multifocal | 2/6 | 2/6 |
| **URINARY BLADDER (N)** | 6/6 | 6/6 |
| **SPLEEN (N)** | 6/6 | 6/6 |
| **THYMUS (N)** | 2/6 | 3/6 |
| Age-related involution | 2/6 | 2/6 |
| Absent* | 2/6 | 1/6 |
| **MESENTERIC LYMPHNODES (N)** | 6/6 | 6/6 |
| **PITUITARY GLAND (N)** | 5/6 | 4/6 |
| Vacuolation, pars distalis | 1/6 | 1/6 |
| Cyst, pars distalis | 0/6 | 1/6 |
| **OVARIES (N)** | 5/6 | 6/6 |
| Cyst, follicular, focal | 1/6 | 0/6 |
| **ADRENAL GLANDS (N)** | 6/6 | 5/6 |
| Cyst, cortex | 0/6 | 1/6 |
| **UTERUS with cervix and vagina (N)** | 5/6 | 6/6 |
| Dilatation, glandular, cystic, multifocal | 1/6 | 0/6 |
| **HEART (N)** | 6/6 | 6/6 |
| **AORTA (N)** | 6/6 | 6/6 |
| **SALIVARY GLANDS (N)** | 6/6 | 5/6 |
| Infiltration, PMNC, multifocal | 0/6 | 1/6 |
| **MANDIBULAR LYMPHNODES (N)** | 6/6 | 5/6 |
| Infiltration, PMNC, multifocal | 0/6 | 1/6 |
| **ESOPHAGUS (N)** | 6/6 | 6/6 |
| **TRACHEA (N)** | 6/6 | 6/6 |
| **THYROID (N)** | 3/6 | 3/6 |
| Cystic follicle, focal | 3/6 | 3/6 |
| **PARATHYROID (N)** | 6/6 | 6/6 |

**Key:** N –within normal histological limits; MNC – Mononuclear Cell; PMNC – Polymorpho-nuclear cell; * - Thymus was absent or replaced with fat might be due to age related complete involution

**Supplementary Table 14: Continued**

**Gender: Female No. of Animals/Group/Sex: 6**

| **Organs/**  **Histopathology** |  | |
| --- | --- | --- |
|  | **G4** | **G6** |
| **STOMACH (N)** | 5/6 | 5/6 |
| Infiltration, MNC, multifocal | 1/6 | 1/6 |
| **PANCREAS (N)** | 6/6 | 5/6 |
| Infiltration, MNC, multifocal, mesentery | 0/6 | 1/6 |
| **DUODENUM (N)** | 6/6 | 6/6 |
| **JEJUNUM (N)** | 6/6 | 6/6 |
| **ILEUM with Peyer’s patches (N)** | 6/6 | 6/6 |
| **CECUM (N)** | 6/6 | 6/6 |
| **COLON (N)** | 6/6 | 6/6 |
| **RECTUM (N)** | 6/6 | 6/6 |
| **BRAIN (N)** | 6/6 | 6/6 |
| **SCIATIC NERVE (N)** | 6/6 | 6/6 |
| **EYES with optic nerve (N)** | 6/6 | 6/6 |
| **SKIN (N)** | 6/6 | 6/6 |
| **MAMMARY GLANDS (N)** | 6/6 | 6/6 |
| **SKELETAL MUSCLE (N)** | 6/6 | 6/6 |
| **SPINAL CORD (N)** | 6/6 | 6/6 |
| **FEMUR WITH JOINT (N)** | 6/6 | 6/6 |
| **SITE OF INJECTION (N)** | 6/6 | 6/6 |

**Key:** N –within normal histological limits; MNC – Mononuclear Cell; PMNC – Polymorpho-nuclear cell.

**Supplementary Table 14: Continued**

**Gender: Male No. of Animals/Group/Sex: 6**

| **Organs/**  **Histopathology** |  | |
| --- | --- | --- |
|  | **G1** | **G3** |
| **Bone Marrow Smear (N)** | 6/6 | 6/6 |

**Gender: Female No. of Animals/Group/Sex: 6 Refer Appendix: 13**

| **Organs/**  **Histopathology** |  | |
| --- | --- | --- |
|  | **G1** | **G3** |
| **Bone Marrow Smear (N)** | 6/6 | 5/5 |

Key: N –within normal histological limits

**Supplementary Table** **15: Summary of Electrocardiogram Value in the animal study iii (in dogs).**

**Gender: Male**

| **Parameters** | **Time Point (0 min)** | | | | | | | | |
| --- | --- | --- | --- | --- | --- | --- | --- | --- | --- |
|  | **G1-Placebo** | | | **G2-Vaccine 1X** | | | **G3-Vaccine 3X** | | |
|  | **Mean** | **SD** | **N** | **Mean** | **Mean** | **SD** | **N** | **Mean** | **Mean** |
| **RR** | 698.21 | 215.55 | 3 | 646.87 | 68.82 | 3 | 661.36 | 130.53 | 3 |
| **HR** | 91.96 | 29.75 | 3 | 93.42 | 9.37 | 3 | 93.40 | 20.42 | 3 |
| **PR** | 107.49 | 9.55 | 3 | 95.73 | 7.10 | 3 | 98.70 | 5.63 | 3 |
| **QRS** | 46.50 | 4.10 | 3 | 43.73 | 1.77 | 3 | 41.27 | 3.98 | 3 |
| **QT** | 219.64 | 19.95 | 3 | 209.85 | 6.36 | 3 | 216.71 | 8.60 | 3 |
| **QTcF** | 253.13 | 3.43 | 3 | 246.26 | 3.85 | 3 | 254.05 | 16.90 | 3 |

| **Parameters** | **Time Point (5 min)** | | | | | | | | |
| --- | --- | --- | --- | --- | --- | --- | --- | --- | --- |
|  | **G1-Placebo** | | | **G2-Vaccine 1X** | | | **G3-Vaccine 3X** | | |
|  | **Mean** | **SD** | **N** | **Mean** | **SD** | **N** | **Mean** | **SD** | **N** |
| **RR** | 754.19 | 154.07 | 3 | 597.61 | 29.59 | 3 | 688.73 | 93.08 | 3 |
| **HR** | 81.79 | 16.52 | 3 | 100.56 | 4.86 | 3 | 88.27 | 12.81 | 3 |
| **PR** | 108.93 | 4.38 | 3 | 89.90↓ | 6.76 | 3 | 98.38 | 8.13 | 3 |
| **QRS** | 47.12 | 3.68 | 3 | 44.89 | 2.81 | 3 | 41.01 | 3.56 | 3 |
| **QT** | 227.77 | 14.47 | 3 | 206.24 | 10.36 | 3 | 215.39 | 5.97 | 3 |
| **QTcF** | 255.13 | 6.24 | 3 | 248.32 | 10.19 | 3 | 248.67 | 5.20 | 3 |

| **Parameters** | **Time Point (10 min)** | | | | | | | | |
| --- | --- | --- | --- | --- | --- | --- | --- | --- | --- |
|  | **G1-Placebo** | | | **G2-Vaccine 1X** | | | **G3-Vaccine 3X** | | |
|  | **Mean** | **SD** | **N** | **Mean** | **SD** | **N** | **Mean** | **SD** | **N** |
| **RR** | 737.94 | 92.34 | 3 | 686.05 | 83.22 | 3 | 723.99 | 128.85 | 3 |
| **HR** | 82.14 | 10.04 | 3 | 88.27 | 10.01 | 3 | 84.79 | 16.23 | 3 |
| **PR** | 110.81 | 3.55 | 3 | 93.19↓↓ | 5.10 | 3 | 99.81 | 6.14 | 3 |
| **QRS** | 47.26 | 3.74 | 3 | 43.59 | 1.87 | 3 | 40.49 | 4.29 | 3 |
| **QT** | 233.31 | 12.96 | 3 | 211.33↓ | 5.59 | 3 | 217.53 | 4.36 | 3 |
| **QTcF** | 262.75 | 11.36 | 3 | 242.97 | 1.30 | 3 | 247.64 | 10.15 | 3 |

| **Parameters** | **Time Point (15 min)** | | | | | | | | |
| --- | --- | --- | --- | --- | --- | --- | --- | --- | --- |
|  | **G1-Placebo** | | | **G2-Vaccine 1X** | | | **G3-Vaccine 3X** | | |
|  | **Mean** | **SD** | **N** | **Mean** | **SD** | **N** | **Mean** | **SD** | **N** |
| **RR** | 784.52 | 88.71 | 3 | 769.97 | 106.75 | 3 | 755.58 | 89.55 | 3 |
| **HR** | 77.10 | 8.21 | 3 | 78.87 | 10.23 | 3 | 80.20 | 9.96 | 3 |
| **PR** | 112.10 | 4.32 | 3 | 96.41↓ | 4.98 | 3 | 101.03 | 8.35 | 3 |
| **QRS** | 46.61 | 3.21 | 3 | 43.99 | 2.26 | 3 | 41.77 | 0.60 | 3 |
| **QT** | 236.47 | 15.32 | 3 | 219.18 | 10.77 | 3 | 225.29 | 6.29 | 3 |
| **QTcF** | 261.25 | 10.28 | 3 | 243.86 | 3.80 | 3 | 253.07 | 19.03 | 3 |

**Key:** N= Number of animals, ↓= significantly low as compared to G1 at p≤0.05, ↓↓= significantly low as compared to G1 at p ≤0.01.

**Supplementary Table 15. Continued**

**Gender: Female**

| **Parameters** | **Time Point (0 min)** | | | | | | | | |
| --- | --- | --- | --- | --- | --- | --- | --- | --- | --- |
|  | **G1-Placebo** | | | **G2-Vaccine 1X** | | | **G3-Vaccine 3X** | | |
|  | **Mean** | **SD** | **N** | **Mean** | **SD** | **N** | **Mean** | **SD** | **N** |
| **RR** | 556.85 | 72.67 | 3 | 543.65 | 74.29 | 3 | 540.61 | 43.54 | 3 |
| **HR** | 108.90 | 13.21 | 3 | 111.72 | 14.92 | 3 | 111.49 | 9.42 | 3 |
| **PR** | 99.96 | 7.26 | 3 | 104.05 | 3.41 | 3 | 105.97 | 13.31 | 3 |
| **QRS** | 47.08 | 7.26 | 3 | 43.52 | 4.50 | 3 | 47.17 | 5.17 | 3 |
| **QT** | 217.30 | 13.28 | 3 | 210.45 | 10.58 | 3 | 205.05 | 10.70 | 3 |
| **QTcF** | 266.35 | 6.38 | 3 | 260.49 | 3.39 | 3 | 253.75 | 8.42 | 3 |

| **Parameters** | **Time Point (5 min)** | | | | | | | | |
| --- | --- | --- | --- | --- | --- | --- | --- | --- | --- |
|  | **G1-Placebo** | | | **G2-Vaccine 1X** | | | **G3-Vaccine 3X** | | |
|  | **Mean** | **SD** | **N** | **Mean** | **SD** | **N** | **Mean** | **SD** | **N** |
| **RR** | 586.41 | 89.47 | 3 | 554.72 | 38.10 | 3 | 521.11 | 75.86 | 3 |
| **HR** | 103.82 | 14.82 | 3 | 108.51 | 7.56 | 3 | 116.71 | 16.25 | 3 |
| **PR** | 103.11 | 7.95 | 3 | 99.82 | 7.53 | 3 | 103.53 | 16.60 | 3 |
| **QRS** | 46.56 | 6.05 | 3 | 43.22 | 1.72 | 3 | 46.21 | 4.20 | 3 |
| **QT** | 224.59 | 10.49 | 3 | 216.92 | 7.66 | 3 | 204.90 | 14.75 | 3 |
| **QTcF** | 270.64 | 2.37 | 3 | 266.39 | 4.82 | 3 | 257.13 | 7.42 | 3 |

| **Parameters** | **Time Point (10 min)** | | | | | | | | |
| --- | --- | --- | --- | --- | --- | --- | --- | --- | --- |
|  | **G1-Placebo** | | | **G2-Vaccine 1X** | | | **G3-Vaccine 3X** | | |
|  | **Mean** | **SD** | **N** | **Mean** | **SD** | **N** | **Mean** | **SD** | **N** |
| **RR** | 573.95 | 43.69 | 3 | 610.43 | 70.08 | 3 | 522.89 | 37.48 | 3 |
| **HR** | 104.95 | 8.12 | 3 | 99.15 | 11.21 | 3 | 115.13 | 7.94 | 3 |
| **PR** | 102.39 | 8.09 | 3 | 103.14 | 7.78 | 3 | 99.79 | 11.55 | 3 |
| **QRS** | 46.73 | 6.28 | 3 | 44.96 | 3.82 | 3 | 45.33 | 4.90 | 3 |
| **QT** | 224.83 | 7.63 | 3 | 223.76 | 8.95 | 3 | 204.75↓ | 3.40 | 3 |
| **QTcF** | 272.49 | 4.98 | 3 | 267.29 | 2.58 | 3 | 256.02↓ | 7.79 | 3 |

| **Parameters** | **Time Point (15 min)** | | | | | | | | |
| --- | --- | --- | --- | --- | --- | --- | --- | --- | --- |
|  | **G1-Placebo** | | | **G2-Vaccine 1X** | | | **G3-Vaccine 3X** | | |
|  | **Mean** | **SD** | **N** | **Mean** | **SD** | **N** | **Mean** | **SD** | **N** |
| **RR** | 651.16 | 45.34 | 3 | 690.33 | 35.35 | 3 | 541.28↓ | 15.47 | 3 |
| **HR** | 92.45 | 6.70 | 3 | 87.07 | 4.47 | 3 | 110.91↓↓ | 3.18 | 3 |
| **PR** | 102.06 | 7.46 | 3 | 103.67 | 9.37 | 3 | 101.45 | 13.47 | 3 |
| **QRS** | 47.07 | 6.58 | 3 | 46.03 | 3.75 | 3 | 44.16 | 4.64 | 3 |
| **QT** | 233.93 | 0.34 | 3 | 235.69 | 8.80 | 3 | 209.03↓↓ | 6.45 | 3 |
| **QTcF** | 273.89 | 5.52 | 3 | 271.76 | 5.92 | 3 | 258.70 | 9.92 | 3 |

**Key:** N= Number of animals, ↓= significantly low as compared to G1 at p≤0.05, ↓↓= significantly low as compared to G1 at p ≤0.01.

**Supplementary Table** **16. Summary of Haematology Parameters**

**Gender: Male**

| **Parameters** | **Group & Dose** | | | | | | | | |
| --- | --- | --- | --- | --- | --- | --- | --- | --- | --- |
|  | **G1-Placebo** | | | **G2-Vaccine 1X** | | | **G3-Vaccine 3X** | | |
|  | **Mean** | **SD** | **N** | **Mean** | **SD** | **N** | **Mean** | **SD** | **N** |
| RBC (10^6^cells/µl) | 6.88 | 0.60 | 3 | 6.31 | 0.40 | 3 | 6.00 | 0.32 | 3 |
| HGB (g/dl) | 15.77 | 0.97 | 3 | 14.40 | 0.95 | 3 | 13.70 | 0.61 | 3 |
| HCT (%) | 45.80 | 2.57 | 3 | 42.93 | 1.93 | 3 | 41.10 | 1.74 | 3 |
| MCV (fl) | 66.70 | 2.09 | 3 | 68.10 | 1.28 | 3 | 68.57 | 1.03 | 3 |
| MCH (pg) | 22.97 | 0.72 | 3 | 22.80 | 0.20 | 3 | 22.93 | 0.25 | 3 |
| MCHC (g/dl) | 34.40 | 0.20 | 3 | 33.50↓ | 0.62 | 3 | 33.43↓ | 0.12 | 3 |
| RDW (%) | 12.77 | 0.15 | 3 | 12.90 | 0.26 | 3 | 13.13 | 0.57 | 3 |
| RET(x10^9^ cells/l) | 7.30 | 4.33 | 3 | 13.77 | 2.10 | 3 | 7.27 | 1.50 | 3 |
| PLT (10^3^cells/µl) | 324.33 | 33.86 | 3 | 584.33↑↑ | 61.85 | 3 | 359.33 | 78.37 | 3 |
| MPV (fl) | 9.20 | 0.78 | 3 | 8.33 | 0.21 | 3 | 8.20 | 0.44 | 3 |
| WBC (10^3^cells/µl) | 8.47 | 0.06 | 3 | 7.87 | 0.37 | 3 | 7.18 | 1.03 | 3 |
| NEU (10^3^cells/µl) | 5.70 | 0.33 | 3 | 5.07 | 0.53 | 3 | 4.14 | 1.43 | 3 |
| LYM (10^3^cells/µl) | 2.02 | 0.01 | 3 | 2.06 | 0.27 | 3 | 2.08 | 0.24 | 3 |
| MON (10^3^cells/µl) | 0.27 | 0.06 | 3 | 0.33 | 0.06 | 3 | 0.36 | 0.04 | 3 |
| EOS (10^3^cells/µl) | 0.38 | 0.19 | 3 | 0.28 | 0.08 | 3 | 0.45 | 0.24 | 3 |
| BAS (10^3^cells/µl) | 0.03 | 0.01 | 3 | 0.04 | 0.01 | 3 | 0.03 | 0.01 | 3 |
| NEU(%) | 67.30 | 3.55 | 3 | 64.33 | 4.54 | 3 | 56.53 | 12.53 | 3 |
| LYM(%) | 23.83 | 0.25 | 3 | 26.23 | 3.88 | 3 | 29.73 | 7.86 | 3 |
| MON(%) | 3.17 | 0.81 | 3 | 4.17 | 0.81 | 3 | 5.03↑ | 0.29 | 3 |
| EOS(%) | 4.53 | 2.23 | 3 | 3.53 | 1.10 | 3 | 6.70 | 4.60 | 3 |
| BAS(%) | 0.37 | 0.06 | 3 | 0.50 | 0.10 | 3 | 0.47 | 0.06 | 3 |

**Key:** N= Number of animals, ↓= significantly low as compared to G1 at p ≤0.05, ↑↑= significantly high as compared to G1 at p≤0.01.

**Supplementary Table 16. Continued**

**Gender: Female**

| **Parameters** | **Group** | | | | | | | | |
| --- | --- | --- | --- | --- | --- | --- | --- | --- | --- |
|  | **G1-Placebo** | | | **G2-Vaccine 1X** | | | **G3-Vaccine 3X** | | |
|  | **Mean** | **SD** | **N** | **Mean** | **SD** | **N** | **Mean** | **SD** | **N** |
| RBC (10^6^cells/µl) | 5.31 | 0.65 | 3 | 6.66 | 0.98 | 3 | 6.09 | 0.28 | 3 |
| HGB (g/dl) | 11.93 | 1.33 | 3 | 15.50 | 2.35 | 3 | 13.97 | 0.97 | 3 |
| HCT (%) | 35.13 | 4.23 | 3 | 46.07 | 6.67 | 3 | 41.47 | 2.94 | 3 |
| MCV (fl) | 66.10 | 1.85 | 3 | 69.23 | 1.63 | 3 | 68.00 | 2.00 | 3 |
| MCH (pg) | 22.47 | 0.40 | 3 | 23.27 | 0.15 | 3 | 22.93 | 0.64 | 3 |
| MCHC (g/dl) | 33.97 | 0.47 | 3 | 33.63 | 0.65 | 3 | 33.73 | 0.06 | 3 |
| RDW (%) | 13.50 | 0.62 | 3 | 12.80 | 0.66 | 3 | 13.37 | 0.93 | 3 |
| RET(x10^9^ cells/l) | 6.17 | 1.82 | 3 | 15.07 | 8.90 | 3 | 11.37 | 9.90 | 3 |
| PLT (10^3^cells/µl) | 513.67 | 195.77 | 3 | 452.33 | 152.40 | 3 | 431.33 | 150.44 | 3 |
| MPV (fl) | 9.37 | 0.45 | 3 | 8.83 | 0.42 | 3 | 7.70↓↓ | 0.26 | 3 |
| WBC (10^3^cells/µl) | 7.95 | 0.54 | 3 | 9.98 | 1.91 | 3 | 7.42 | 0.74 | 3 |
| NEU (10^3^cells/µl) | 5.02 | 0.86 | 3 | 6.42 | 1.79 | 3 | 4.86 | 0.24 | 3 |
| LYM (10^3^cells/µl) | 2.08 | 0.29 | 3 | 2.65 | 0.25 | 3 | 1.90 | 0.48 | 3 |
| MON (10^3^cells/µl) | 0.52 | 0.09 | 3 | 0.44 | 0.10 | 3 | 0.27↓ | 0.03 | 3 |
| EOS (10^3^cells/µl) | 0.15 | 0.06 | 3 | 0.33 | 0.12 | 3 | 0.27 | 0.15 | 3 |
| BAS (10^3^cells/µl) | 0.03 | 0.02 | 3 | 0.03 | 0.00 | 3 | 0.03 | 0.01 | 3 |
| NEU(%) | 62.87 | 7.48 | 3 | 63.60 | 6.68 | 3 | 65.93 | 4.97 | 3 |
| LYM(%) | 26.27 | 4.42 | 3 | 27.17 | 5.31 | 3 | 25.30 | 4.17 | 3 |
| MON(%) | 6.60 | 1.57 | 3 | 4.40 | 0.85 | 3 | 3.67 | 0.75 | 3 |
| EOS(%) | 1.97 | 0.93 | 3 | 3.30 | 1.05 | 3 | 3.53 | 1.79 | 3 |
| BAS(%) | 0.37 | 0.21 | 3 | 0.33 | 0.06 | 3 | 0.37 | 0.12 | 3 |

**Key:** N= Number of animals, ↓= significantly low as compared to G1 at p≤0.05, ↓↓= significantly low as compared to G1 at p≤0.01.

**Supplementary Table 17**. **Summary of Coagulation Parameters in the animal study iii (in dogs).**

**Gender: Male**

| ***Parameters*** | **Group** | | | | | | | | |
| --- | --- | --- | --- | --- | --- | --- | --- | --- | --- |
|  | **G1-Placebo** | | | **G2-Vaccine 1X** | | | **G3-Vaccine 3X** | | |
|  | **Mean** | **SD** | **N** | **Mean** | **SD** | **N** | **Mean** | **SD** | **N** |
| **PT _(Sec)_** | 8.37 | 0.15 | 3 | 8.07 | 0.81 | 3 | 8.73 | 0.12 | 3 |
| **APTT _(Sec)_** | 9.57 | 1.46 | 3 | 9.40 | 0.82 | 3 | 10.27 | 0.49 | 3 |

**Gender: Female**

| ***Parameters*** | **Group & Dose** | | | | | | | | |
| --- | --- | --- | --- | --- | --- | --- | --- | --- | --- |
|  | **G1-Placebo Control** | | | **G2-Vaccine 1X** | | | **G3-Vaccine 3X** | | |
|  | **Mean** | **SD** | **N** | **Mean** | **SD** | **N** | **Mean** | **SD** | **N** |
| **PT _(Sec)_** | 8.13 | 0.49 | 3 | 8.10 | 0.46 | 3 | 9.27 | 0.78 | 3 |
| **APTT _(Sec)_** | 10.40 | 0.89 | 3 | 10.07 | 0.31 | 3 | 11.67 | 1.85 | 3 |

**Supplementary Table 18. Summary of Clinical Chemistry Parameters in the animal study iii (in dogs).**

**Gender: Male**

| **Parameters** | **Group & Dose** | | | | | | | | |
| --- | --- | --- | --- | --- | --- | --- | --- | --- | --- |
|  | **G1-Placebo Control** | | | **G2-Vaccine 1X** | | | **G3-Vaccine 3X** | | |
|  | **Mean** | **SD** | **N** | **Mean** | **SD** | **N** | **Mean** | **SD** | **N** |
| GLU (mg/dl) | 101.67 | 2.08 | 3 | 92.00↓↓ | 1.00 | 3 | 107.00 | 5.00 | 3 |
| TP (g/dl) | 5.83 | 0.21 | 3 | 6.03 | 0.12 | 3 | 6.63↑ | 0.40 | 3 |
| ALB (g/dl) | 3.20 | 0.17 | 3 | 3.37 | 0.21 | 3 | 3.40 | 0.30 | 3 |
| GLO (g/dl) | 2.57 | 0.12 | 3 | 2.70 | 0.17 | 3 | 3.27↑ | 0.31 | 3 |
| A/G Ratio | 1.23 | 0.15 | 3 | 1.27 | 0.15 | 3 | 1.07 | 0.12 | 3 |
| TRI (mg/dl) | 39.67 | 6.43 | 3 | 46.67 | 4.62 | 3 | 46.33 | 6.43 | 3 |
| CHO(mg/dl) | 208.02 | 10.19 | 3 | 204.78 | 14.38 | 3 | 198.86 | 31.91 | 3 |
| ALT (U/l) | 58.00 | 19.31 | 3 | 65.00 | 33.06 | 3 | 47.67 | 7.37 | 3 |
| AST (U/l) | 34.67 | 11.72 | 3 | 40.33 | 11.02 | 3 | 37.33 | 4.04 | 3 |
| ALP (U/l) | 106.33 | 33.02 | 3 | 140.67 | 43.59 | 3 | 166.67 | 86.41 | 3 |
| GGT (U/l) | 16.00 | - | 1 | 13.00 | 1.41 | 3 | 13.00 | 1.73 | 3 |
| BIL (mg/dl) | - | - | - | 0.15 | 0.07 | 2 | 0.4 | - | 1 |
| BUN (mg/dl) | 9.67 | 0.58 | 3 | 11.33 | 1.15 | 3 | 8.67 | 2.52 | 3 |
| CRE (mg/dl) | 0.67 | 0.06 | 3 | 0.63 | 0.06 | 3 | 0.67 | 0.06 | 3 |
| Na (mmol/l) | 138.67 | 1.15 | 3 | 139.33 | 0.58 | 3 | 139.33 | 0.58 | 3 |
| K (mmol/l) | 4.43 | 0.15 | 3 | 4.60 | 0.17 | 3 | 4.27 | 0.35 | 3 |
| Cl (mmol/l) | 113.67 | 0.58 | 3 | 116.67↑ | 0.58 | 3 | 114.67 | 1.53 | 3 |
| Ca (mg/dl) | 10.40 | 0.36 | 3 | 10.50 | 0.10 | 3 | 10.63 | 0.15 | 3 |
| P (mg/dl) | 4.40 | 1.25 | 3 | 4.83 | 0.23 | 3 | 4.27 | 0.23 | 3 |

**Key:** N= Number of animals, ↑= significantly high as compared G1 at p≤0.05, ↓↓= significantly low as compared to G1 at p≤0.01.

**Supplementary Table 18. Continued**

**Gender: Female Refer Appendix: 10**

| **Parameters** | **Group & Dose** | | | | | | | | |
| --- | --- | --- | --- | --- | --- | --- | --- | --- | --- |
|  | **G1-Placebo Control** | | | **G2-Vaccine 1X** | | | **G3-Vaccine 3X** | | |
|  | **Mean** | **SD** | **N** | **Mean** | **SD** | **N** | **Mean** | **SD** | **N** |
| GLU (mg/dl) | 105.00 | 12.12 | 3 | 91.67 | 7.37 | 3 | 102.33 | 2.52 | 3 |
| TP (g/dl) | 6.47 | 1.06 | 3 | 6.40 | 0.44 | 3 | 6.23 | 0.42 | 3 |
| ALB (g/dl) | 3.33 | 0.55 | 3 | 3.67 | 0.45 | 3 | 3.57 | 0.29 | 3 |
| GLO (g/dl) | 3.10 | 0.61 | 3 | 2.73 | 0.15 | 3 | 2.67 | 0.15 | 3 |
| A/G Ratio | 1.10 | 0.17 | 3 | 1.33 | 0.23 | 3 | 1.37 | 0.06 | 3 |
| TRI (mg/dl) | 96.67 | 46.00 | 3 | 67.67 | 19.30 | 3 | 67.67 | 39.40 | 3 |
| CHO (mg/dl) | 334.38 | 86.14 | 3 | 281.03 | 51.32 | 3 | 291.55 | 91.19 | 3 |
| ALT (U/l) | 84.33 | 83.10 | 3 | 61.67 | 23.18 | 3 | 45.33 | 4.93 | 3 |
| AST (U/l) | 30.67 | 5.69 | 3 | 39.00 | 2.00 | 3 | 38.00 | 3.61 | 3 |
| ALP (U/l) | 170.67 | 30.62 | 3 | 178.67 | 63.58 | 3 | 123.00 | 2.00 | 3 |
| GGT(U/l) | - | - | - | 11.50 | 0.71 | 2 | 12.50 | 2.12 | 2 |
| BIL (mg/dl) | - | - | - | 0.40 | 0.28 | 2 | 0.25 | 0.21 | 2 |
| BUN (mg/dl) | 7.67 | 1.53 | 3 | 9.00 | 1.73 | 3 | 8.00 | 1.00 | 3 |
| CRE (mg/dl) | 0.47 | 0.06 | 3 | 0.53 | 0.15 | 3 | 0.50 | 0.17 | 3 |
| Na (mmol/l) | 138.33 | 2.52 | 3 | 139.33 | 1.53 | 3 | 137.33 | 1.53 | 3 |
| K (mmol/l) | 4.53 | 0.12 | 3 | 4.77 | 0.12 | 3 | 4.37 | 0.25 | 3 |
| Cl (mmol/l) | 112.67 | 2.08 | 3 | 116.33 | 3.51 | 3 | 113.33 | 3.51 | 3 |
| Ca (mg/dl) | 11.20 | 1.28 | 3 | 11.00 | 0.26 | 3 | 10.93 | 0.35 | 3 |
| P (mg/dl) | 5.70 | 0.26 | 3 | 4.93 | 0.38 | 3 | 4.17↓↓ | 0.55 | 3 |

**Key:** N= No. of Animals, ↓↓= significantly low as compared to G1 at p≤0.01.

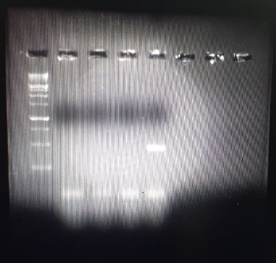
Full gel picture of the above DNA gel.

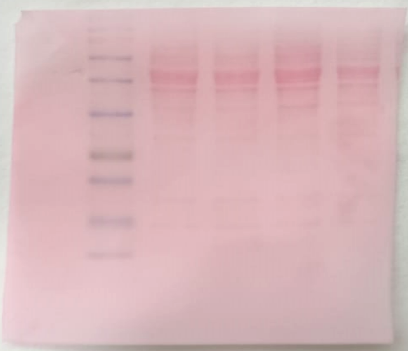


Full gel and blot images of above

Western blot data.
